# Engineered Multifunctional Hydrogel Delivering Novel CBX7 Inhibitor Modulates Cuproptosis Via Liquid–Liquid Phase Separation to Restore Cardiac Function in Aged Myocardial Infarction

**DOI:** 10.1002/advs.202511630

**Published:** 2025-10-21

**Authors:** Jun Liu, Peng Qu, Jiao Shi, Tingting Liang, Yao Gu, Xue Li, Cui Ma, Danyang Zhao, Feila Liu, Qi Liang, Panke Cheng, Qian Lei

**Affiliations:** ^1^ Department of Clinical Laboratory Affiliated Hospital of North Sichuan Medical College Nanchong 637000 China; ^2^ School of Laboratory Medicine North Sichuan Medical College Nanchong 637007 China; ^3^ Translational Medicine Research Center North Sichuan Medical College Nanchong 637007 China; ^4^ Institute of Cardiovascular Diseases & Department of Cardiology Sichuan Provincial People's Hospital School of Medicine University of Electronic Science and Technology of China Chengdu 610072 China; ^5^ Department of Mathematics Army Medical University Chongqing 400038 China; ^6^ Department of Anesthesiology Sichuan Provincial People's Hospital School of Medicine University of Electronic Science and Technology of China Chengdu 610072 China; ^7^ School of Pharmacy and Bioengineering Chongqing University of Technology Chongqing 400050 China; ^8^ Ultrasound Medicine and Computational Cardiology Key Laboratory of Sichuan Province Chengdu 610072 China

**Keywords:** CBX7, cuproptosis, hydrogel, liquid–liquid phase separation, δ‐Amyrenone

## Abstract

Cardiac repair after myocardial ischemia–reperfusion (MIR) declines with aging. This study shows that Chromobox 7 (CBX7) acts in an age‐dependent manner, in young hearts, it promotes cardiomyocyte proliferation, whereas in aged hearts, CBX7 forms liquid–liquid phase separation (LLPS) with ATP7A, trapping ATP7A intracellularly, reducing membrane trafficking and copper efflux, and triggering cuproptosis. High‐throughput screening identifies δ‐Amyrenone (δAe) as a selective CBX7 inhibitor that disrupts CBX7‐ATP7A LLPS, restores ATP7A trafficking and copper efflux, and improves cardiac function while reducing fibrosis and arrhythmias. Single‐cell RNA‐seq shows MIR‐induced cuproptosis‐related loss is concentrated in NR4A3 positive cardiomyocytes and RGCC positive capillary endothelial cells in aged hearts. To enhance delivery, this study engineered a multifunctional conductive hydrogel with antioxidant, pro‐angiogenic, immunomodulatory, O_2_ releasing and electrical properties. Loaded with δAe, this single‐injection hydrogel provides controlled release, alleviates cuproptosis‐related mitochondrial injury, and pairs its intrinsic repair capacity with CBX7 inhibition to drive ATP7A trafficking, enhance copper efflux, and suppress cuproptosis. In aged mouse and Bama minipig MIR models, this strategy improves structural, functional, and electrophysiological outcomes, supporting translational potential.

## Graphical abstract

An age‐adapted therapy pairs δ‐Amyrenone, a CBX7 inhibitor, with an alginate hydrogel (CSAδ) to rescue infarcted elderly hearts. CSAδ disrupts CBX7–ATP7A liquid–liquid phase separation, restores ATP7A trafficking and copper efflux, suppresses cuproptosis, supplies oxygen, scavenges ROS, promotes M2 polarization, supports angiogenesis, and enhances electrical coupling. Efficacy and safety validated in aged mice and Bama minipigs.

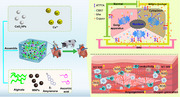

## 1 Introduction

The rate of adult cardiomyocyte turnover in mammals significantly declines with age,^[^ ^1^ ^]^ and the reduction in regenerative capacity is primarily attributed to cell cycle exit following differentiation, changes in metabolic pathways, and alterations in chromatin structure.^[^ ^2^ ^]^ Previous studies have shown that modulating key molecular targets and their associated signaling pathways can promote cardiomyocyte regeneration in mature hearts.^[^ ^3^ ^]^ For example, inhibition of Hippo or activation of YAP in the Hippo‐YAP pathway allows cardiomyocytes to re‐enter the cell cycle and proliferate;^[^ ^4^ ^]^ activation of the Wnt/β‐catenin signaling also promotes proliferation;^[^ ^5^ ^]^ while activating the ERBB2‐Neuregulin pathway not only promotes cardiomyocyte regeneration but also induces epithelial‐mesenchymal transition‐like responses, which facilitate cardiac repair.^[^ ^6^ ^]^ On the other hand, studies have found that Chromobox 7(CBX7) protein inhibits cardiomyocyte proliferation via the TARDBP/RBM38/p21 pathway, preventing re‐entry into the cell cycle.^[^ ^7^ ^]^ In a mouse model of myocardial ischemia–reperfusion (MIR), CBX7 knockout significantly enhanced cardiomyocyte proliferation, suggesting that CBX7 is an important regulatory target for myocardial regeneration.^[^ ^7^ ^]^ However, most studies on CBX7 have focused on young adult mice, whereas MIR predominantly occurs in the elderly population. Consider that the same protein can exhibit functional heterogeneity across different age stages (for example, in youth, YAP mainly promotes the expression of cell cycle genes to drive proliferation and tissue growth, while in older individuals, it tends to activate stress resistance pathways, such as antioxidant responses and metabolic adaptation;^[^ ^8^, ^9^ ^]^ similarly, Sestrin family proteins coordinate proliferation and metabolism in youth, but after aging, they delay cell degeneration by inhibiting reactive oxygen species (ROS) accumulation, maintaining mitochondrial homeostasis, and regulating autophagy).^[^ ^10^ ^]^ Therefore, it remains unclear whether CBX7 has a similar regulatory effect in aged hearts and whether its mechanism can promote recovery after elderly MIR, which warrants further investigation. Current protein intervention strategies include small molecule compounds, monoclonal antibodies, RNA interference, gene editing (such as CRISPR‐Cas), and recombinant protein therapies. Among these, small‐molecule compounds are promising in clinical applications due to their low manufacturing costs, excellent tissue penetration, and convenience for oral administration.^[^ ^11^ ^]^ Small molecule screening mainly relies on high‐throughput screening and virtual docking technologies; hence, identifying high‐affinity CBX7 inhibitors for MIR therapy has become a highly focused research direction. In addition, liquid–liquid phase separation (LLPS), an emerging intracellular regulatory mechanism, forms dynamic “droplets” through intermolecular interactions, aiding the aggregation of metabolic enzymes and optimizing cellular responses under stress conditions. For example, the glycolytic enzymes PKM2 and PFK1 can undergo LLPS and regulate glycolytic flux and efficiency in specific regions.^[^ ^12^, ^13^ ^]^ However, it is still unclear whether CBX7 in the cytoplasm participates in LLPS and how its interaction with other proteins affects cellular function. On the other hand, the mechanism of copper‐induced cell death (cuproptosis) has garnered attention in recent years. Its core involves the abnormal binding of copper ions with acetylated proteins in the mitochondria, triggering protein aggregation, metabolic dysregulation, and redox imbalance, ultimately leading to cell death.^[^ ^14^, ^15^ ^]^ Studies have found that the copper ion content in local myocardial tissue increases after MIR, with this effect potentially being more pronounced in elderly patients.^[^ ^16^ ^]^ Therefore, investigating whether copper‐induced cell death damages aged cardiomyocytes and whether regulating this process can offer myocardial protection has become an urgent issue to address. In terms of drug delivery strategies, small molecule drugs are currently mainly administered via intravenous injection, but they have drawbacks, such as short half‐lives, rapid metabolism and clearance, non‐specific distribution leading to toxicity, poor targeting, and the need for frequent administration.^[^ ^17^ ^]^ Although targeted nanomaterials have reduced off‐target organ damage to some extent, their targeting efficiency still needs improvement.^[^ ^18^ ^]^ Therefore, developing drug delivery systems with controlled release properties, such as hydrogels, has become a key direction to enhance efficacy and safety. Percutaneous coronary intervention (PCI), a standard treatment for MIR, provides feasibility for local slow‐release hydrogel injection via catheter during PCI. Existing hydrogels mainly focus on single functions (e.g., ROS clearance, electrical coupling improvement, and angiogenesis regulation),^[^ ^19^, ^20^, ^21^ ^]^ but given the complex microenvironment following MIR, characterized by exacerbated inflammation, excessive ROS, local hypoxia, impaired angiogenesis, and inadequate electrical coupling, there is an urgent need to develop a multifunctional integrated regulatory system.

**Scheme 1:**
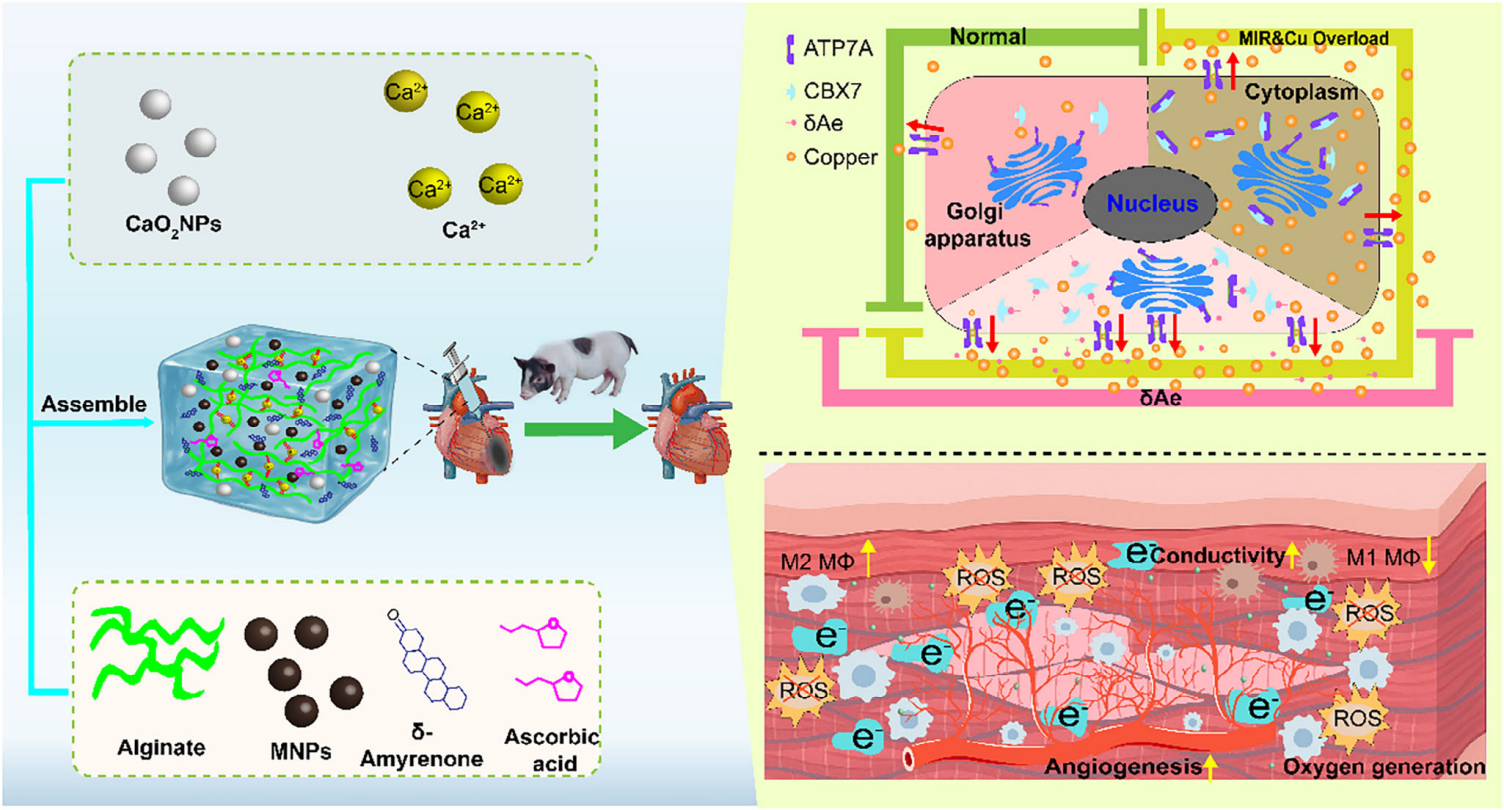
δAe‐loaded multifunctional conductive hydrogel targets the CBX7‐ATP7A LLPS to repair the aged MIR heart. Left. Hydrogel formulation and assembly. An alginate matrix is ionically crosslinked by Ca^2^⁺ and doped with calcium peroxide nanoparticles (CaO_2_ NPs), melanin nanoparticles (MNPs), δ‐Amyrenone (δAe), and ascorbic acid (AsA), assembled into an integrated, multifunctional platform, administered by intramyocardial injection in mice and Bama minipigs. Upper right. Cellular mechanism. Under basal conditions, ATP7A traffics to the membrane to mediate copper efflux. In aged MIR with copper overload, CBX7 undergoes LLPS with ATP7A, retaining ATP7A intracellularly, impairing efflux, and triggering cuproptosis. δAe disrupts CBX7‐ATP7A LLPS, restores ATP7A membrane translocation and copper efflux, and suppresses cuproptosis. Lower right. Tissue‐level synergy. The multifunctional hydrogel provides sustained oxygen release, promotes angiogenesis, scavenges excess ROS, shifts macrophages toward the M2 phenotype, O_2_ releasing, and improves electrical coupling. These effects, together with in situ controlled release of δAe, collectively enhance structural remodeling, cardiac function, and electrophysiology. Abbreviations. MIR, myocardial ischemia/reperfusion; LLPS, liquid–liquid phase separation; MNPs, melanin nanoparticles; ROS, reactive oxygen species.

Despite recent advances in biomaterials for post–MIR repair, most approaches target single pathways: oxygen‐releasing hydrogels risk early burst and pH perturbation, ROS scavengers have short half‐lives and limited coverage of reactive species, immunomodulatory patches or drug delivery cannot simultaneously address hypoxia and copper imbalance, copper chelation/metal‐homeostasis strategies are constrained by systemic toxicity and specificity; and cell/exosome therapies suffer from batch variability and poor homing. To tackle the multiaxis dysregulation in aged MIR hearts, this study posited and tested a central hypothesis: CBX7 exerts age‐divergent functions, and in aged myocardium, it undergoes LLPS with ATP7A, impeding its membrane trafficking, diminishing copper efflux, and precipitating cuproptosis. Guided by this axis, this study used high‐throughput screening to identify δAe as a selective CBX7 inhibitor and defined its age‐dependent actions‐promoting cardiomyocyte cell‐cycle re‐entry in young hearts while, in aged cardiomyocytes, blocking CBX7‐ATP7A LLPS to restore copper homeostasis. further engineered an injectable, conductive, sustained‐release hydrogel for in situ delivery and microenvironmental co‐modulation, integrating temporally controlled δAe release with oxygen supplementation, broad ROS buffering, pro‐angiogenic cues, immunomodulation, and improved electrical coupling. Anchored on the CBX7‐ATP7A‐copper efflux‐cuproptosis axis, this work combines single‐cell transcriptomics with aged mouse and Bama minipig MIR models to evaluate the strategy from molecular and cellular mechanisms to tissue structure and electrophysiology, outlining a mechanism‐driven, materials‐enabled path toward precise control of copper homeostasis in the aging heart (**Scheme** **1**).

## 2 Results

### 2.1 CBX7 Knockout Protects the Aged Heart After MIR

**Figure 1:**
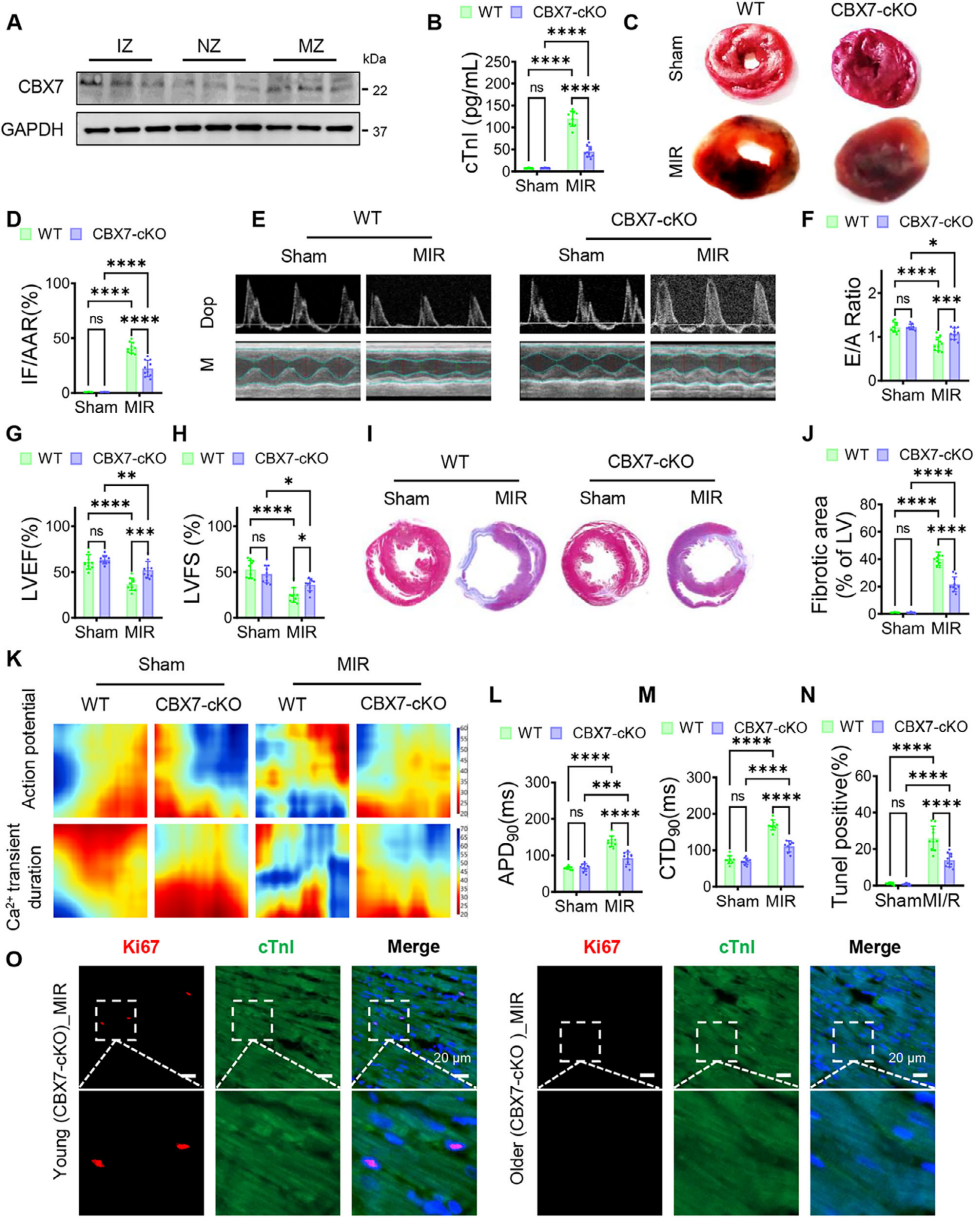
CBX7 knockout mitigates injury and electrical remodeling in aged MIR hearts. Aged mice underwent myocardial ischemia/reperfusion (MIR) with or without cardiomyocyte‐specific CBX7 deletion (CBX7‐cKO). Readouts cover acute injury (24 h), subacute tissue remodeling, and functional‐electrical outcomes (up to 4 weeks). A) Western blots show CBX7 upregulation after MIR, most prominently in the infarct zone (IZ) relative to the marginal (MZ) and normal zones (NZ). B) Serum cTnI at 24 h indicates reduced myonecrosis in CBX7‐cKO versus control. C,D) TTC‐stained short‐axis sections and quantification (infarct size normalized to area at risk, IF/AAR) demonstrate a smaller infarct burden with CBX7 loss. E–H) Representative Doppler (top) and M‐mode (bottom) echocardiograms with corresponding quantification show improved diastolic filling (E/A) and systolic performance (LVEF, LVFS) in CBX7‐cKO. I,J) Masson's trichrome staining reveals attenuated collagen deposition; fibrosis (% LV) is reduced in CBX7‐cKO, consistent with favorable post‐MIR remodeling. K–M) Optical mapping heatmaps and pooled metrics indicate that CBX7‐cKO suppresses MIR‐induced prolongation of action potentials and Ca^2^⁺ transients, with shorter APD90 and CTD90 toward physiological ranges, features associated with lower arrhythmic susceptibility and improved excitation–contraction coupling. N) TUNEL quantification at 24 h shows fewer apoptotic nuclei in CBX7‐cKO myocardium. O) Immunofluorescence in young versus aged CBX7‐cKO hearts after MIR (Ki67, red; cTnI, green; DAPI, blue; scale bar, 20 µm) illustrates regenerative heterogeneity: robust Ki67⁺ cardiomyocytes in young hearts but minimal proliferation in aged hearts, indicating that protection in aged CBX7‐cKO hearts is achieved largely via survival and remodeling rather than myocyte cell‐cycle re‐entry. Data are mean ± SD (*n* = 10 per group unless noted). one‐way ANOVA with Tukey post hoc test. ^*^ *p* < 0.05, ^**^ *p* < 0.01, ^***^ *p* < 0.005, ^****^ *p* < 0.001; ns, not significant. Abbreviations. MIR, myocardial ischemia reperfusion; IZ, infarct zone; MZ, marginal zone; NZ, normal zone; IF/AAR, infarct size over area at risk; E/A, early/late mitral inflow ratio; LVEF, left ventricular ejection fraction; LVFS, LV fractional shortening; APD90, action potential duration at 90% repolarization; CTD90, Ca^2^⁺‐transient duration to 90% recovery; LV, left ventricle.

At 1 day after MIR, CBX7 was markedly upregulated in aged mouse hearts at the mRNA and protein levels, with higher protein abundance in the infarct and marginal zones versus the normal zone (**Figure** **1**A; Figure S1A,B, Supporting Information). This study, therefore tested whether cardiomyocyte‐specific CBX7 deletion (CBX7‐cKO) affords protection in aged MIR. Compared with wild‐type (WT), CBX7‐cKO significantly reduced acute myocardial injury, evidenced by lower serum cTnI, smaller infarct size on TTC, and decreased CK‐MB (Figure 1B–D; Figure S2A,B, Supporting Information). Echocardiography at 4 weeks showed improved diastolic filling (E/A) and enhanced systolic performance (LVEF, LVFS) without changes in heart rate (Figure 1E–H; Figure S2C, Supporting Information). Histology revealed attenuated fibrosis (Figure 1I,J). Optical mapping demonstrated that CBX7 deletion shortened action potential duration (APD90) and Ca^2+^ transient duration (CTD90), indicating reduced repolarization delay and improved excitation‐contraction coupling (Figure 1K–M; Figure S2D,E, Supporting Information). TUNEL staining confirmed fewer apoptotic/degenerating cardiomyocytes in CBX7‐cKO hearts (Figure 1N; Figure S2F, Supporting Information). Given reports that CBX7 deletion can promote cardiomyocyte proliferation in adults, this study interrogated this in aged mice: Ki67/cTnI co‐staining revealed regenerative activity in young but not aged CBX7‐cKO hearts after MIR (Figure 1O). Thus, in aged myocardium, the benefit of CBX7 knockout arises independent of proliferation, instead aligning with reduced injury, fibrosis, repolarization heterogeneity, and arrhythmia risk.

### 2.2 Identification of a CBX7 Inhibitor and Age‐Dependent Actions of δAe

**Figure 2:**
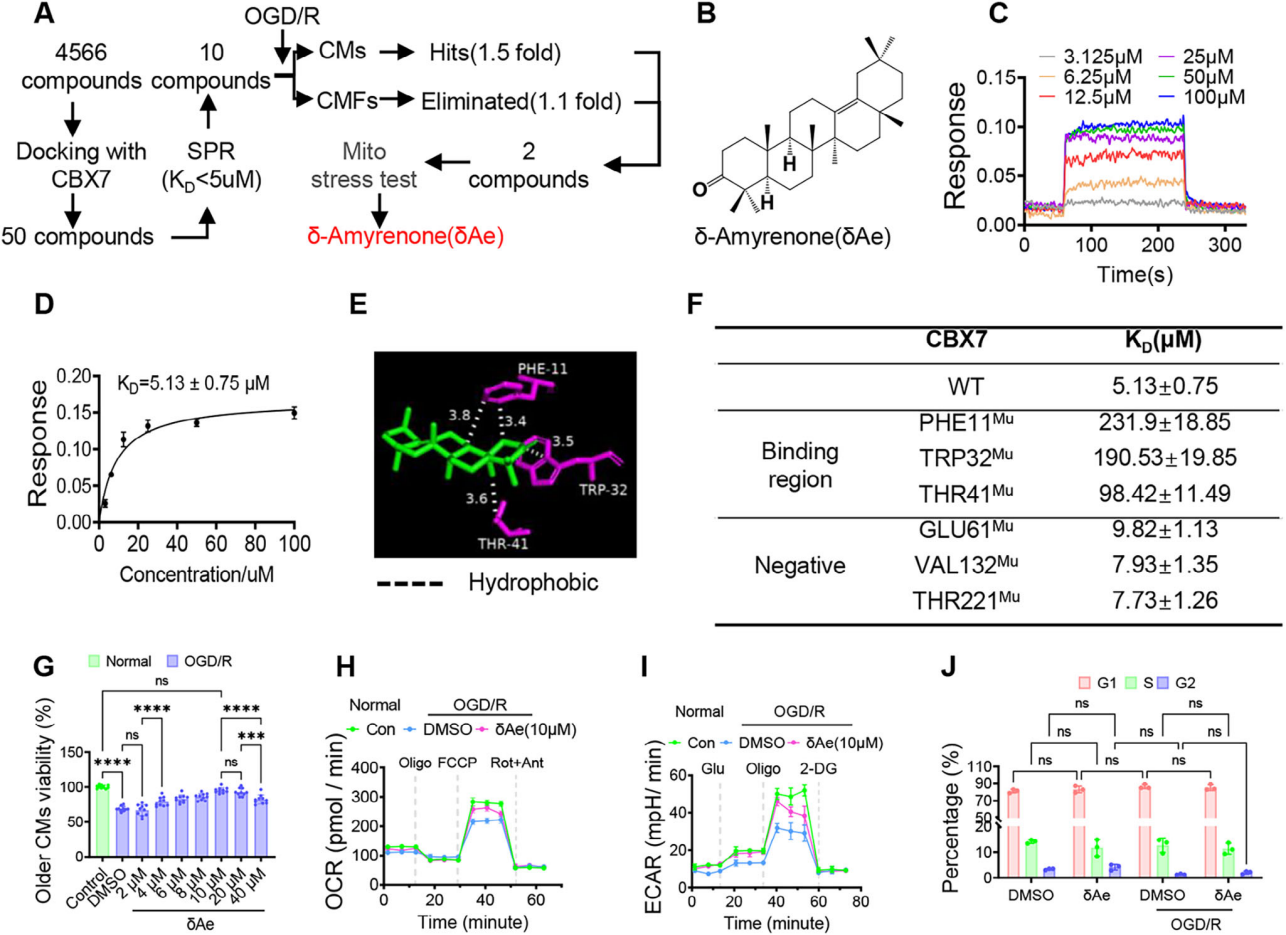
Discovery of δAe as a CBX7 inhibitor and its age‐dependent cellular effects. A) Integrated screening workflow: structure‐guided docking, surface plasmon resonance (SPR) pre‐screening, ischemia‐mimetic testing in adult cardiomyocytes (CMs) and cardiac fibroblasts (CMFs), and mitochondrial stress profiling identified δ‐Amyrenone (δAe) as the lead CBX7 binder with cellular activity under injury stress. B) Chemical structure of δAe. C) SPR sensograms demonstrate rapid association and dissociation with clear concentration dependence for δAe binding to CBX7. D) Global fitting of SPR data yields K_D_ = 5.13 ± 0.75 µm. E) Docking model positions δAe within a CBX7 pocket, forming hydrogen bonds with PHE11, TRP32, and THR41 (3.8, 3.4, and 3.6 Å), indicating a defined interaction motif. F) Mutational mapping by SPR: substitutions at PHE11, TRP32, and THR41 markedly increase K_D_ (reduced affinity), whereas GLU61, VAL132, and THR221 have minor effects, identifying PHE11/TRP32/THR41 as principal contact sites. G) Dose–response under oxygen–glucose deprivation and reoxygenation (OGD/R) identifies 10 µm δAe as non‐toxic and protective; this dose is used for subsequent assays. H) Seahorse oxygen consumption rate (OCR) traces with sequential additions‐oligomycin, then FCCP, then rotenone plus antimycin A, show that δAe partially restores maximal respiratory capacity after OGD/R, indicating preserved mitochondrial reserve. I) Extracellular acidification rate (ECAR) traces with sequential additions‐glucose, then oligomycin, then 2‐deoxy‐D‐glucose, show that δAe improves glycolytic capacity and mitigates OGD/R‐induced glycolytic dysfunction. J) Cell‐cycle analysis in aged CMs shows no significant shift in phase distribution with δAe after OGD/R, indicating that protection in aged cells is achieved primarily through bioenergetic rescue rather than proliferation (in contrast to young CMs, where δAe promotes cell‐cycle re‐entry and reduces apoptosis). Data are mean ± SD from independent experiments (sample sizes in panels). One‐way ANOVA with Tukey's post hoc test. ^***^ *p* < 0.005, ^****^ *p* < 0.001; ns, not significant. Abbreviations. SPR, surface plasmon resonance; OGD/R, oxygen–glucose deprivation and reoxygenation; OCR, oxygen consumption rate; ECAR, extracellular acidification rate; CMs, cardiomyocytes; CMFs, cardiac fibroblasts.

Motivated by the protection conferred by CBX7 knockout, this study conducted a tiered screen to discover small‐molecule inhibitors (**Figure** **2**A). From docking to SPR prefiltering, and then ischemia‐mimetic assays and mitochondrial stress tests, δ‐Amyrenone (δAe) emerged as a lead (Figure 2B). SPR sensograms showed rapid, concentration‐dependent association/dissociation between δAe and CBX7, global fitting yielded a K_D_ of 5.13 ± 0.75 µm (Figure 2C,D). Docking predicted that δAe fits a CBX7 pocket and forms hydrogen bonds with PHE11, TRP32, and THR41 (3.8–3.4–3.6 Å range; Figure 2E). Site‐directed mutagenesis followed by SPR validated these contacts: PHE11^Mu^, TRP32^Mu^, and THR41^Mu^ markedly weakened binding (higher K_D_), whereas GLU61^Mu^, VAL132^Mu^, and THR221^Mu^ had a limited impact (Figure 2F). Functionally, δAe (10 µm) was non‐toxic to aged cardiomyocytes and partially restored bioenergetics after OGD/R, maximal respiration/OCR and glycolytic capacity/ECAR improved versus vehicle (Figure 2G–I; Figure S3A,B, Supporting Information). By contrast, δAe did not alter the cell‐cycle distribution in aged cells (Figure 2J; Figure S3C,D, Supporting Information), indicating a protection mode independent of proliferation. In young cardiomyocytes, δAe increased survival and reduced apoptosis under OGD/R, reversed apoptosis‐related markers (PARP cleavage, Bax balance), enriched the “Cell Cycle” gene set, and promoted G1–S progression with upregulation of CDK2, Cyclin A2 as well as Aldh7a1, FOXM1, and SIRT7 (Figure S4A–M, Supporting Information). Together, δAe is a direct CBX7 binder with defined contact residues, conferring metabolic/mitochondrial protection in aged cells while promoting cell‐cycle re‐entry in young cells‐consistent with an age‐dependent pharmacology.

### 2.3 CBX7‐ATP7A LLPS Restrain Copper Efflux in Aged Cardiomyocytes and are Disrupted by δAe

**Figure 3:**
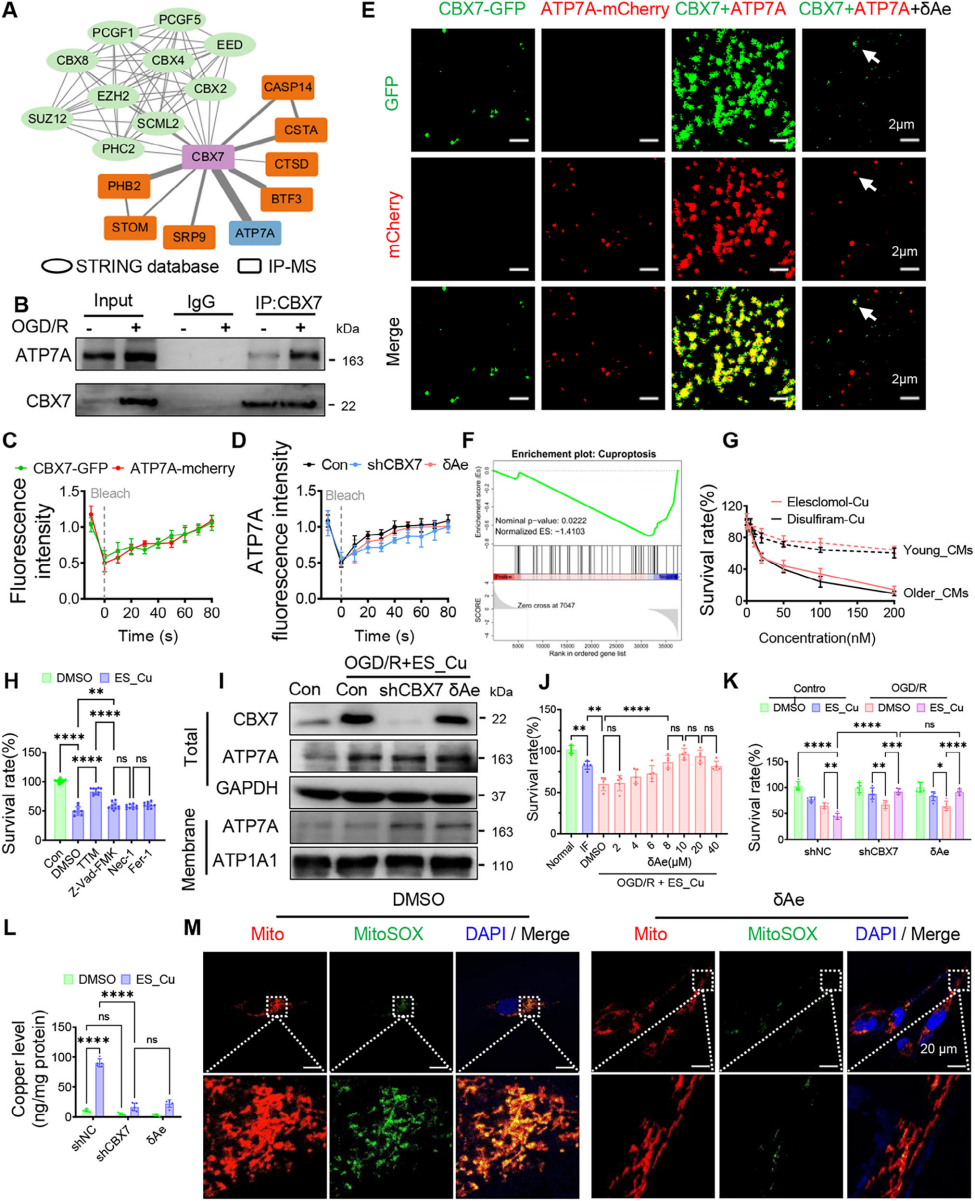
CBX7‐ATP7A LLPS restrains ATP7A trafficking and copper efflux, δAe disrupts condensates and protects aged cardiomyocytes. Aged mouse cardiomyocytes were subjected to oxygen–glucose deprivation and reoxygenation (OGD/R) with genetic or pharmacologic CBX7 perturbation to define how CBX7 controls ATP7A behavior, copper efflux, and cuproptosis. A) CBX7 interactome integrating STRING predictions with immunoprecipitation–mass spectrometry (IP–MS) highlights ATP7A among top partners, alongside canonical PRC members, metabolism, and membrane‐structural proteins. B) Co‐immunoprecipitation confirms CBX7‐ATP7A binding at baseline and after OGD/R (input and IgG controls shown), indicating a stable interaction under stress. C) Fluorescence recovery after photobleaching (FRAP) of CBX7‐GFP and ATP7A‐mCherry demonstrates coordinated, rapid fluorescence recovery, consistent with liquid‐like dynamics. D) Quantified FRAP kinetics show slower ATP7A recovery when CBX7 is silenced (shCBX7) or when cells are treated with δAe, indicating that CBX7 abundance and δAe both modulate the dynamics of the CBX7‐ATP7A condensate. E) In vitro droplet assays reveal liquid‐like droplets when CBX7 and ATP7A are combined, whereas δAe markedly reduces droplet formation (scale bars, 2 µm), supporting a direct effect on condensate assembly. F) Gene set enrichment analysis (GSEA) shows downregulation of a cuproptosis gene set after δAe under OGD/R stress (normalized enrichment score and nominal *p*‐value provided), indicating pathway suppression. G) Copper‐dependent cytotoxicity curves demonstrate greater sensitivity in aged versus young cardiomyocytes to Elesclomol–Cu and Disulfiram–Cu, establishing age‐biased vulnerability to copper stress. H) In aged cardiomyocytes treated with OGD/R plus Elesclomol–Cu at 50 nm, viability rescue is strongest with TTM (a cuproptosis inhibitor), with smaller effects from Z‐VAD‐FMK (apoptosis inhibitor), Nec‐1 (necroptosis inhibitor), and Fer‐1 (ferroptosis inhibitor), indicating that cuproptosis is the dominant death program in this context. I) Western blots of total and membrane fractions show that OGD/R with Elesclomol–Cu elevates total ATP7A; shCBX7 and δAe increase ATP7A in the membrane fraction (controls: GAPDH for cytosol, ATP1A1 for membrane), consistent with restored membrane trafficking required for copper efflux. J) δAe dose‐response under OGD/R with Elesclomol–Cu identifies low‐micromolar concentrations that improve viability without toxicity. K) Across matched conditions, shCBX7 and δAe both increase cell survival, with additive protection seen against copper stress. L) Cellular copper quantification shows lower intracellular copper with shCBX7 or δAe relative to controls, consistent with enhanced efflux. (M) MitoSOX imaging shows reduced mitochondrial ROS in shCBX7 and δAe groups compared with OGD/R plus Elesclomol–Cu alone (Mito, red; MitoSOX, green; DAPI, blue; scale bar, 20 µm), linking condensate disruption and ATP7A relocalization to mitochondrial protection. Data are mean ± SD with sample sizes indicated in the panels. One‐way ANOVA with Tukey's post hoc test. ^*^ *p* < 0.05, ^**^ *p* < 0.01, ^***^ *p* < 0.005, ^****^ *p* < 0.001; ns, not significant. Abbreviations. IP–MS, immunoprecipitation–mass spectrometry; OGD/R, oxygen–glucose deprivation and reoxygenation; FRAP, fluorescence recovery after photobleaching; LLPS, liquid–liquid phase separation; ES_Cu, Elesclomol–copper; TTM, tetrathiomolybdate.

To define CBX7's functional partners in aged cardiomyocytes, this study integrated STRING predictions with IP–MS and obtained an interaction map enriched for polycomb repressive complex components as well as metabolism/membrane proteins, with ATP7A ranking prominently (**Figure** **3**A). Co‐immunoprecipitation confirmed CBX7‐ATP7A binding at baseline and after ischemic challenge (Figure 3B). Fluorescence‐recovery analyses revealed coordinated dynamics of CBX7‐GFP and ATP7A‐mCherry (Figure 3C); CBX7 knockdown slowed ATP7A recovery, and δAe similarly altered recovery kinetics, indicating perturbed complex dynamics (Figure 3D). In vitro reconstitution showed CBX7 and ATP7A co‐assemble into liquid‐like droplets that were markedly diminished by δAe (Figure 3E), supporting a LLPS mechanism. Functionally, δAe suppressed a cuproptosis gene set following ischemic stress (NES < 0; Figure 3F). Aged cardiomyocytes were more vulnerable to copper‐dependent death than young cells upon Elesclomol‐Cu or Disulfiram‐Cu exposure (Figure 3G); 50 nm Elesclomol‐Cu (ES_Cu) reduced viability to ≈50% and was used for subsequent assays. Under OGD/R and ES_Cu stimulation, total ATP7A increased, whereas CBX7 knockdown or δAe shifted ATP7A toward the plasma membrane, consistent with restored copper efflux competence (Figure 3I). δAe improved survival in a low‐micromolar window (peak at ≈10 µm; Figure 3J) and conferred protection comparable to CBX7 silencing across conditions (Figure 3K). Correspondingly, δAe lowered intracellular copper burden and mitochondrial ROS (Figure 3L,M). Together, these data indicate that CBX7‐ATP7A LLPS sequesters ATP7A away from the membrane in aged cardiomyocytes, limiting copper export and sensitizing cells to cuproptosis. δAe disrupts these condensates, promotes ATP7A membrane trafficking, and restores copper homeostasis.

### 2.4 Design and Performance of a δAe‐Loadable Multifunctional Conductive Hydrogel

**Figure 4:**
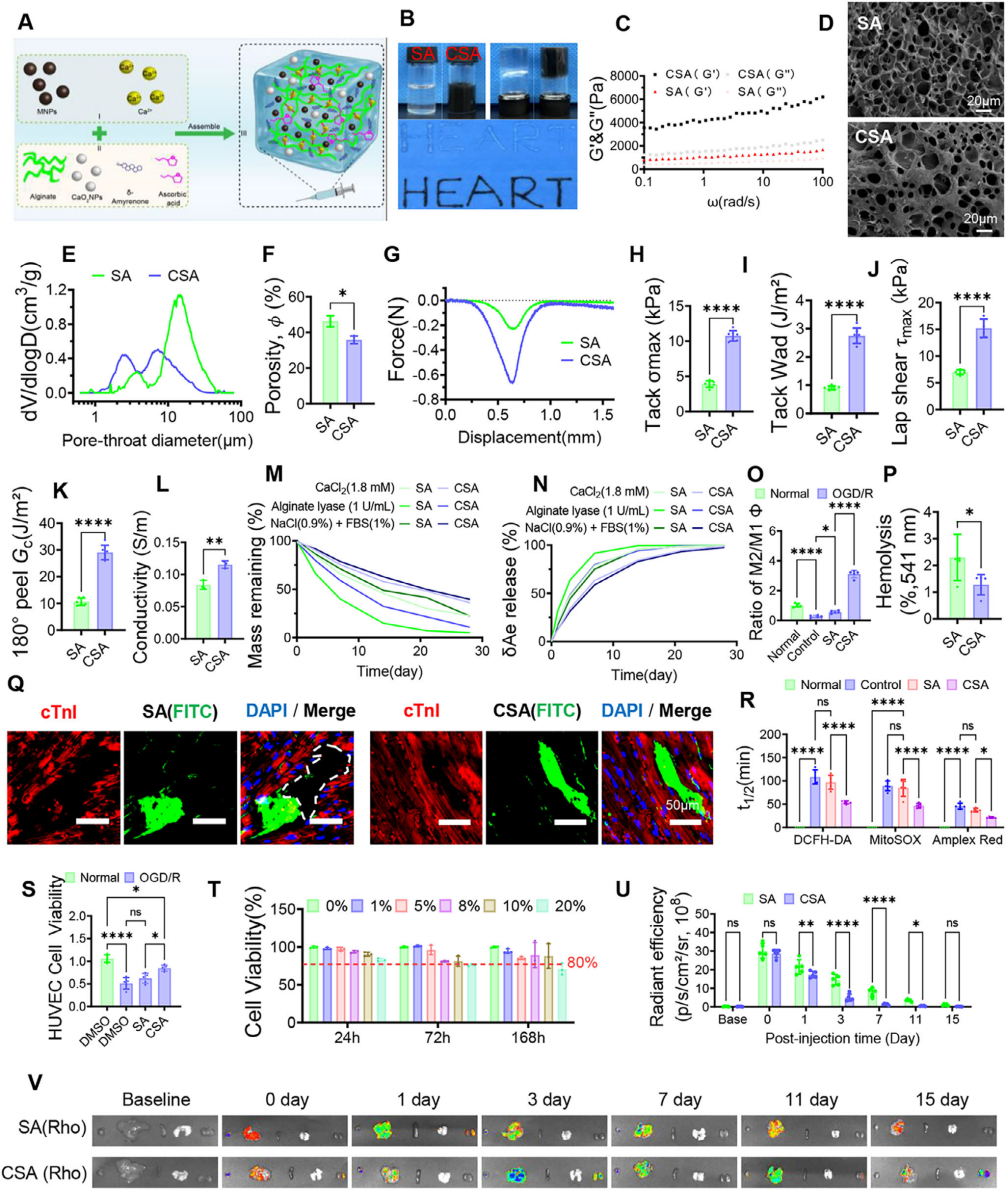
Design and performance of the δAe‐loadable multifunctional hydrogel. A) Assembly scheme of the composite CSA hydrogel: alginate matrix ionically crosslinked by calcium, incorporating calcium peroxide nanoparticles (CaO_2_ NPs) for controlled oxygenation, melanin nanoparticles (MNPs) for broad‐spectrum reactive oxygen species buffering, and ascorbic acid for pH stabilization; CSAδ denotes CSA loaded with δ‐Amyrenone (δAe). B) Macroscopic appearance of gels, illustrating injectability and uniformity. C) Post‐injection rheology (frequency sweep) comparing the storage modulus G′ and loss modulus G″ of SA versus CSA, showing higher G′ and a larger G′–G″ gap in CSA, indicative of a stronger elastic network after in situ gelation. D) Scanning electron microscopy reveals a denser, more interconnected pore network in CSA (scale bars, 20 µm). E,F) Mercury intrusion porosimetry: the differential intrusion curve dV/dlogD shows the principal peak of CSA shifted toward smaller pore‐throat diameters, with corresponding porosity quantification, supporting enhanced mechanical integrity and controlled mass transport. G) Representative tack force–displacement traces illustrating interfacial debonding behavior on cardiac tissue. H) Peak tack stress (σ_max_) and I) work of adhesion (Wad) demonstrate higher instant contact adhesion for CSA. J) Lap‐shear strength (τ_max_) and K) peel energy at one‐hundred‐eighty degrees (Gc) show improved sustained adhesion, supporting stable myocardial fixation under motion and perfusion. L) Electrical conductivity of SA and CSA shows that CSA reaches a physiologically relevant range for myocardial coupling, favoring improved signal transmission across the infarct border. M) Mass remaining over time in relevant media indicates predictable degradation suitable for post‐infarct remodeling timelines. N) Cumulative δAe release from CSAδ demonstrates sustained, near‐linear release over days, enabling local maintenance of therapeutic levels. O) Ratio of M2 to M1 macrophages under oxidative stress conditions increases with CSA, indicating immunomodulation toward a reparative phenotype. P) Hemolysis percentage remains below accepted safety thresholds, confirming blood compatibility. Q) In‐tissue imaging of fluorescein‐labeled gels (FITC) co‐stained with cardiac troponin I (cTnI) shows intimate gel‐myocardium contact without cavity formation at the injection site (scale bars, 50 µm). R) Half‐lives of oxidant readouts, DCFH‐DA (total reactive oxygen species), MitoSOX (mitochondrial superoxide), and Amplex Red (extracellular hydrogen peroxide), are shorter with CSA, indicating faster and more complete scavenging compared with SA. S) Human umbilical vein endothelial cell viability under oxygen and glucose deprivation followed by reoxygenation is higher with CSA, consistent with pro‐angiogenic support in hostile microenvironments. T) Cytocompatibility of SA and CSA across gel concentrations in aged cardiomyocytes at 24, 72, and 168 h confirms a broad safety window. U,V) Near‐infrared imaging of rhodamine‐labeled gels demonstrates greater myocardial retention for CSA compared with SA, shown by higher radiant efficiency and slower signal decay over serial time points. Data are mean ± SD. One‐way analysis of variance with Tukey's post hoc test. ^*^ *p* < 0.05, ^**^ *p* < 0.01, ^***^ *p* < 0.005, ^****^ *p* < 0.001; ns, not significant. Abbreviations. SA, alginate hydrogel; CSA, composite alginate hydrogel with CaO_2_ nanoparticles, melanin nanoparticles, and ascorbic acid; CSAδ, CSA loaded with δ‐Amyrenone; G′, storage modulus; G″, loss modulus; σmax, peak tack stress; Wad, work of adhesion; τmax, peak lap‐shear strength; Gc, peel energy at one‐hundred‐eighty degrees; FITC, fluorescein isothiocyanate; Rho, rhodamine; HUVEC, human umbilical vein endothelial cell.

This study first synthesized a multifunctional alginate/Ca^2^⁺ hydrogel (CSA) by integrating calcium peroxide nanoparticles (CaO_2_; sustained O_2_ release), melanin nanoparticles (MNPs; broad‐spectrum ROS buffering and conductivity enhancement), and ascorbic acid (pH control), and subsequently loaded δ‐Amyrenone (δAe) for localized, sustained delivery (CSAδ) (**Figure** **4**A,B). XRD and SEM confirmed crystalline CaO_2_ and spherical MNPs (Figure S5A,B, Supporting Information). To justify the inclusion of MNPs in a δAe‐containing system, this study directly compared δAe, MNPs, and δAe+MNPs under OGD/R. Across total ROS (DCFH‐DA), mitochondrial ROS (MitoSOX), and extracellular H_2_O_2_ (Amplex Red), the combination produced the fastest and deepest suppression with the shortest half‐lives, outperforming either component alone (Figure S5C–F, Supporting Information). This synergistic control of oxidative stress translated to better mitochondrial integrity (higher JC‐1 and ATP), greater cardiomyocyte viability, lower IL‐1β and TNF‐α, and macrophage polarization toward CD206⁺ (M2) with an increased M2/M1 ratio (Figure S5G–M, Supporting Information). Mechanistically, MNPs rapidly buffer multiple ROS species in the early phase, whereas δAe acts on the mitochondrial‐copper axis to curb later ROS generation, yielding time‐staggered complementarity. These data establish that adding MNPs to a δAe‐based platform is not redundant but necessary for breadth, speed, and durability of antioxidant defense. Having established the component rationale, this study then benchmarked SA (alginate/Ca^2^⁺) against CSA at the materials and functional levels. FTIR confirmed component signatures in the composites (Figure S6A, Supporting Information). Pre‐injection rheology showed shear‐thinning for both precursors (catheter‐compatible; Figure S6B, Supporting Information). Post‐injection, frequency sweeps revealed solid‐like behavior with higher G′/G″ for CSA than SA (Figure 4C). Microstructurally, CSA exhibited a tighter pore–throat distribution and lower porosity (Figure 4D), consistent with mercury intrusion indicating a denser network (Figure 4E,F; Figure S6C, Supporting Information). In wet‐adhesion tests, CSA outperformed SA in tack (σ_max_, Wad), lap‐shear (τ_max_), and 180° peel energy (Gc), with representative traces in Figure S6D (Supporting Information) (Figure 4G–K). CSA also showed higher conductivity approaching the myocardial range and could light an LED (Figure 4L; Figure S6E, Supporting Information). Degradation and release profiles demonstrated controlled mass loss and sustained δAe release, while CaO_2_ provided stable oxygenation without alkaline drift (bulk pH ≈7.1–7.4; Figure 4 M,N; Figure S6F,G, Supporting Information). Hemocompatibility and cytocompatibility were favorable (low hemolysis; high cell viability up to 168 h) (Figure 4P,T). Importantly, tissue integration was evaluated in vivo: FITC‐labeled gels localized within infarcted myocardium, and CSA maintained tight apposition to native tissue without cavity formation, evidence of functional biocompatibility and wet adhesion in situ (Figure 4Q). Longitudinal imaging confirmed greater retention of CSA versus SA (Figure 4U,V). At the cellular and microenvironment level, CSA outperformed SA in lowering total and mitochondrial ROS kinetics while minimally perturbing extracellular H_2_O_2_ signaling; it also supported endothelial survival, pro‐angiogenic markers and promoted M2 polarization (Figure 4O,R,S; Figure S6H–P, Supporting Information). Collectively, CSA integrates injectability, wet adhesion, conductivity, oxygenation, broad ROS buffering, immunomodulation, angiogenic support, and sustained δAe delivery, features tailored to the multifactorial demands of the aged MIR niche.

### 2.5 CSAδ Outperforms SAδ and δAe Monotherapy to Repair MIR Injury in Mice

**Figure 5:**
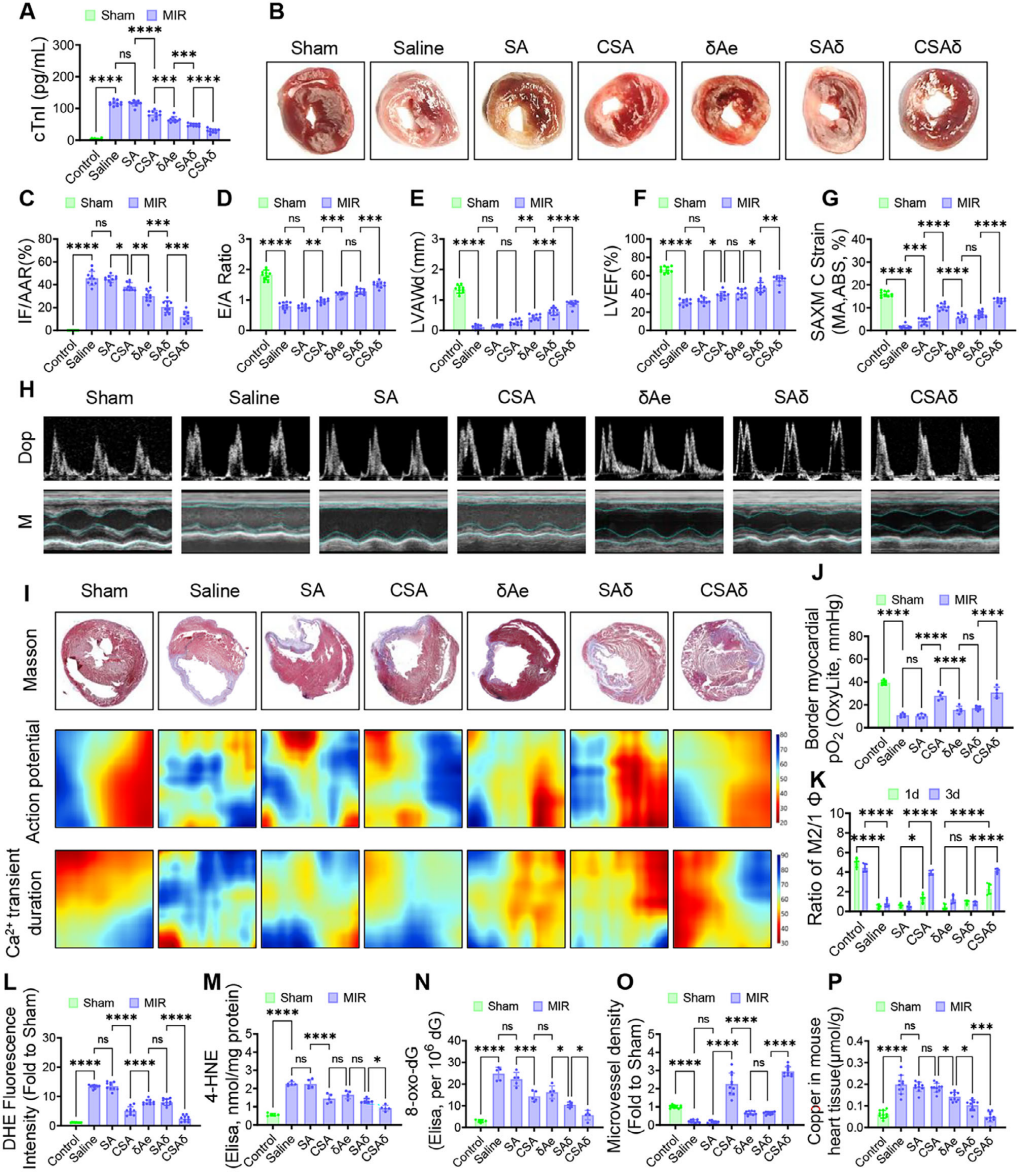
CSAδ improves myocardial repair, milieu, and electrophysiology after MIR in mice. A) Serum cTnI at 24 h. B) TTC‐stained short‐axis sections (representatives). C) IF/AAR (%). D) E/A ratio (Doppler). E) LVAWd (mm; M‐mode). F) LVEF (%). G) Mid–short‐axis circumferential strain (SAXM C strain, %). H) Representative Doppler (top) and M‐mode (bottom) echocardiograms. I) Masson's trichrome (top), action‐potential duration map (middle), and Ca^2^⁺‐transient duration map (bottom). J) Border‐zone pO_2_ (mmHg). K) M2/M1 macrophage ratio at 1 and 3 days. L) DHE fluorescence (ROS). M) 4‐HNE (ELISA). N) 8‐oxo‐dG (ELISA). O) CD31⁺ microvessel density (fold to Sham). P) Myocardial copper content (µmol mg^−1^ protein). Data are mean ± SD (*n* = 10). Two‐way ANOVA with Tukey's post hoc; ^*^ *p*<0.05, ^**^ *p*<0.01, ^***^ *p*<0.005, ^****^ *p*<0.001; ns, not significant. Abbreviations: MIR, myocardial ischemia reperfusion; TTC, 2,3,5‐triphenyltetrazolium chloride; IF/AAR, infarct size over area at risk; LVAWd, LV anterior wall thickness at end‐diastole; LVEF, left ventricular ejection fraction; LVFS, LV fractional shortening; SAXM, short‐axis mid‐level; APD90, action‐potential duration at 90% repolarization; CTD90, Ca^2^⁺‐transient duration to 90% recovery; DHE, dihydroethidium; 4‐HNE, 4‐hydroxynonenal; 8‐oxo‐dG, 8‐oxo‐2′‐deoxyguanosine.

To benchmark in vivo efficacy, mice subjected to MIR were randomized to Sham, Saline, SA, CSA, δAe, SAδ, or CSAδ (*n* = 10/group). CSAδ most strongly reduced acute myocardial injury‐lower serum cTnI and CK‐MB (**Figure** **5**A; Figure S7A, Supporting Information), and limited infarction on TTC (representatives and IF/AAR%; Figure 5B,C; Figure S7B, Supporting Information). Echocardiography showed superior recovery of diastolic filling (E/A), anterior wall thickness (LVAWd), and systolic performance (LVEF, LVFS) with improved mid‐short‐axis circumferential strain (SAXM C strain) (Figure 5D–H; Figure S7C–E, Supporting Information). Fibrosis burden declined in parallel (Figure 5I; Figure S7F, Supporting Information). At the microenvironment level, CSAδ increased border‐zone oxygen tension (pO_2_) and curtailed oxidative stress and damage, lower DHE signal, 4‐HNE, and 8‐oxo‐Dg, while promoting CD31⁺ microvessels (Figure 5J,L–O; Figure S8A, Supporting Information). Immunity shifted toward repair: M2/M1 rose at days 1 and 3, and cardiac TNF‐α/IL‐1β decreased (Figure 5K; Figure S7L and S8A, Supporting Information). Importantly, CSAδ significantly lowered myocardial copper content and suppressed cuproptosis markers versus SAδ and δAe (Figure 5P; Figure S7M, Supporting Information). Optical mapping revealed more favorable electrical remodeling with CSAδ: restored activation window, shortened APD90 and CTD90, normalized Ca^2+^‐transient kinetics, and a lower conduction‐block proportion (Figure 5I; Figure S7G–K, Supporting Information). Heart rate remained unchanged across groups (Figure S7C, Supporting Information). Safety was supported by normal H&E histology of major organs (Figure S8B, Supporting Information). Across injury, function, redox, immune, angiogenic milieu, copper homeostasis, and electrical stability, CSAδ consistently outperformed SAδ and δAe alone, indicating a synergistic benefit from local oxygenation, ROS buffering, pH control, immune reprogramming, and sustained δAe delivery within the conductive adhesive scaffold.

### 2.6 CSAδ Improves Cardiac Structure, Function, and Electrical Stability in Aged Pig MIR Model

**Figure 6:**
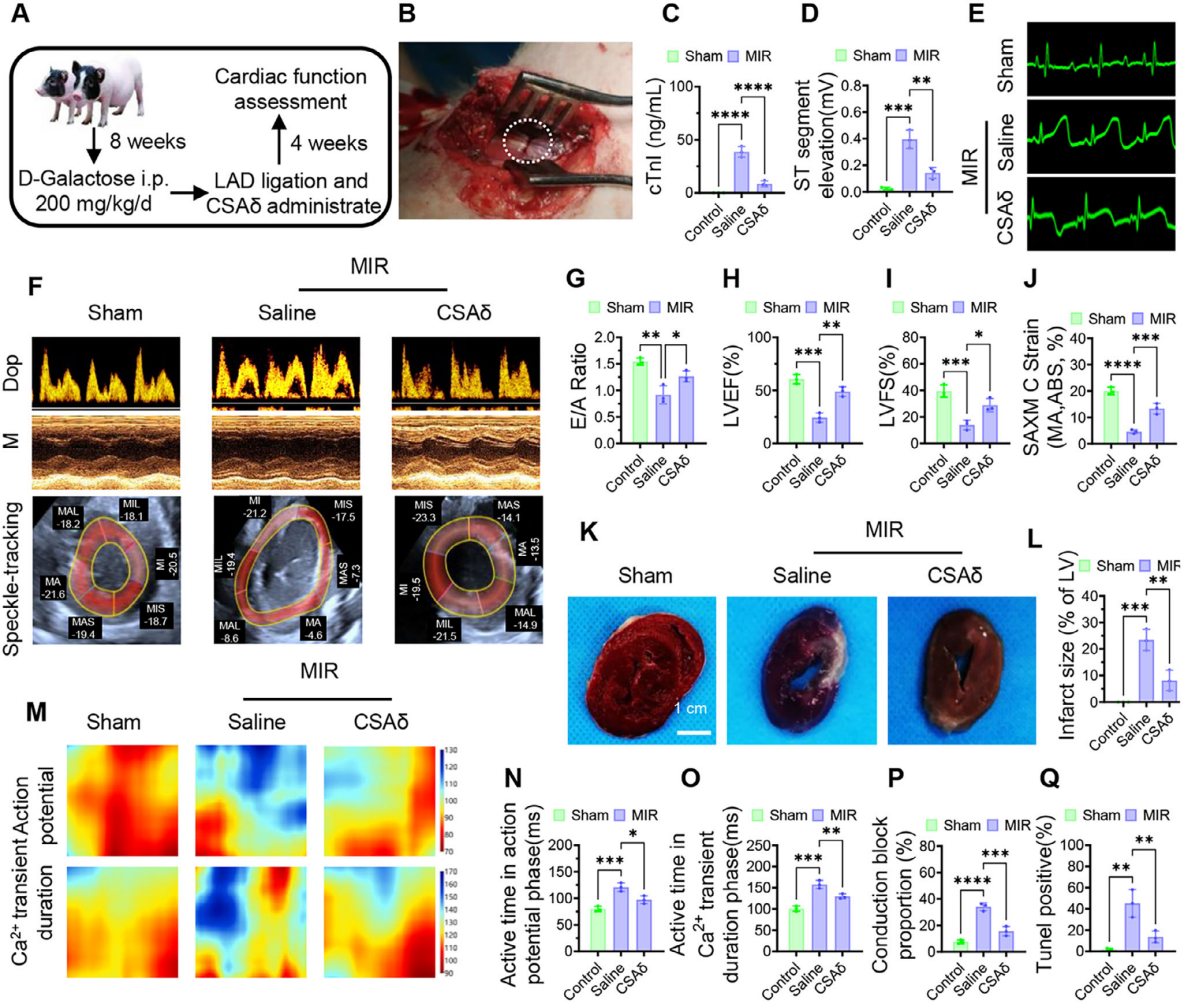
CSAδ restores function and electrical stability in the aged pig MIR model. A) Timeline, Bama miniature pigs were aged with D‐galactose (200 mg kg^−1^ day^−1^, i.p., 8 weeks), then subjected to MIR by transient LAD ligation (45 min) and reperfusion. During reperfusion, animals received intramyocardial saline or CSAδ; Sham underwent thoracotomy without LAD ligation. Follow‐up echocardiography and optical mapping were performed 4 weeks post‐procedure (*n* = 3/group). B) Intraoperative photograph showing LAD ligation site (dotted circle). C) Serum cTnI at 24 h post‐MIR shows lower injury with CSAδ versus saline. D,E) ST‐segment elevation, E) representative ECGs, and D) quantification demonstrate attenuation with CSAδ. Cardiac mechanics and strain. F–J) Echocardiography at 4 weeks, F) Representative Doppler (top), M‐mode (middle), and speckle‐tracking snapshots (bottom; bull's‐eye with mid short‐axis ring), CSAδ improves G) E/A (diastolic filling), H) LVEF, and I) LVFS versus saline, approaching sham levels, J) SAXM circumferential strain (%; absolute values shown), CSAδ restores regional contractility in the infarct ring. K,L) TTC‐stained short‐axis sections K) and quantification of infarct size as %LV L) show ≈65% reduction with CSAδ versus saline. M) Activation and duration maps for action potentials (top) and Ca^2^⁺ transients (bottom). N,O) Active time windows (ms) for action potentials N) and Ca^2^⁺ transients O) indicate faster propagation and recovery with CSAδ. P) Conduction‐block proportion (%) is reduced by CSAδ. Q) TUNEL‐positive cardiomyocytes (%) at 24 h and endpoint are decreased with CSAδ. Data are mean ± SD (*n* = 3). One‐way ANOVA with Tukey's post hoc. ^*^ *p*<0.05, ^**^ *p*<0.01, ^***^ *p*<0.005, ^****^ *p*<0.001. Abbreviations: MIR, myocardial ischemia reperfusion; LAD, left anterior descending coronary artery; ECG, electrocardiogram; cTnI, cardiac troponin I; E/A, early/late mitral inflow; LVEF, left ventricular ejection fraction; LVFS, LV fractional shortening; SAXM, short‐axis mid‐level; TTC, 2,3,5‐triphenyltetrazolium chloride; APD, action‐potential duration; APD90, APD at 90% repolarization; CTD90, Ca^2^⁺‐transient duration to 90% decay; TUNEL, terminal deoxynucleotidyl transferase dUTP nick end labeling.

To approximate human cardiac physiology, this study established an aged Bama miniature pig model by intraperitoneal D‐galactose (200 mg kg^−1^ d^−1^, 8 weeks), which produced oxidative stress, inflammation, and structural degeneration (MDA and IL‐6 up‐expression, SOD and IGF‐1 down‐expression, and reduced LVAWd; Figure S9A–F, Supporting Information). MIR was induced by transient LAD ligation (45 min) and reperfusion (**Figure** **6**A,B). During reperfusion, pigs received saline or CSAδ intramyocardially. CSAδ reduced acute injury: serum cTnI and CK‐MB (24 h) were lower than saline (Figure 6C; Figure S9G, Supporting Information), and ST‐segment elevation was blunted (Figure 6D,E). At 4 weeks, echocardiography showed improved diastolic filling (E/A), systolic function (LVEF, LVFS), and mid‐short‐axis circumferential strain (Figure 6F–J). TTC confirmed a marked reduction in infarct size (Figure 6K,L). Optical mapping demonstrated faster, more homogeneous propagation with shorter activation windows for both action potentials and Ca^2^⁺ transients and fewer conduction blocks (Figure 6M–P); APD90 and CTD90 were also shortened toward physiologic ranges (Figure S9I,J, Supporting Information). Myocardial apoptosis decreased (TUNEL; Figure 6Q; Figure S9K, Supporting Information), while heart rate and systemic liver/kidney indices remained stable (Figure S9H,L–O, Supporting Information). Histology of major organs showed no overt toxicity (Figure S9P, Supporting Information). Together, these results indicate that CSAδ is safe and confers robust benefit in a large‐animal setting, reducing necro‐ischemic injury, restoring chamber mechanics and circumferential strain, and stabilizing electrical conduction in aged pig MIR.

### 2.7 snRNA‐seq Reveals CSAδ Preserves Vulnerable NR4A3^+^CMs and RGCC^+^capECs

**Figure 7:**
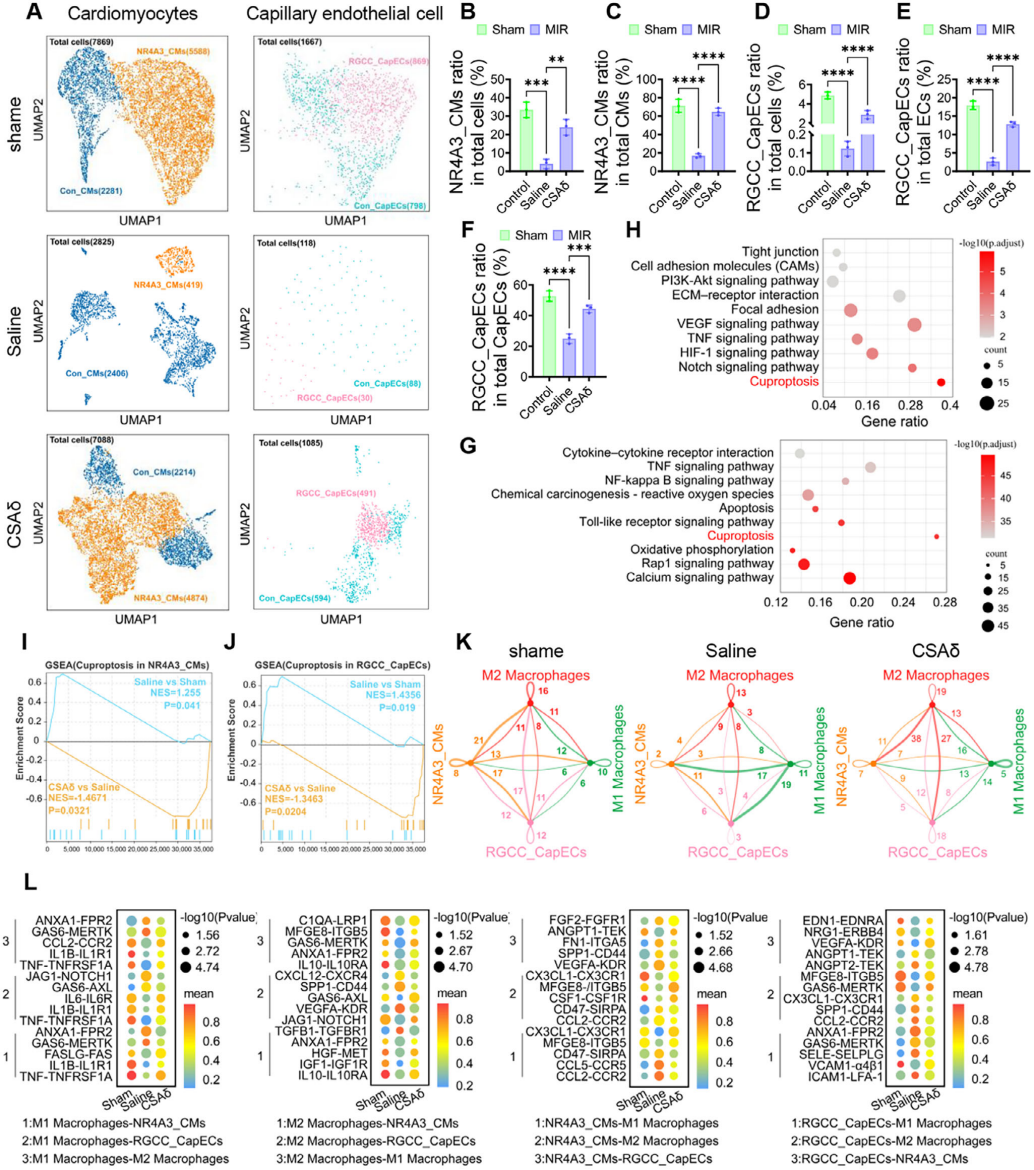
snRNA‐seq in aged pig MIR. Infarct/border myocardium was collected 3 days after MIR from Sham, Saline, and CSAδ pigs. Nuclei were isolated, 10x libraries generated, and datasets QC‐filtered/integrated to yield >2 × 10⁴ high‐quality nuclei spanning major lineages. A) UMAPs for cardiomyocytes (CMs) (left) and capillary endothelial cells (capECs) (right) across groups. The NR4A3_CMs and RGCC_CapECs clusters (yellow, pink) decrease after MIR (Saline) and are preserved with CSAδ. B,C) Ratio of NR4A3_CMs normalized to B) total cells and C) total CMs. D–F) Ratio of RGCC_CapECs normalized to D) total cells, E) total ECs, and F) total capECs. G,H) KEGG enrichment (Saline vs Sham) in G) CMs and H) capECs highlights inflammatory and redox pathways and cuproptosis among top terms. I,J) GSEA of the cuproptosis gene set in I) NR4A3_CMs and J) RGCC_CapECs shows positive enrichment with MIR (Saline vs Sham) and negative enrichment with CSAδ (CSAδ vs Saline), indicating CSAδ suppresses cuproptosis activation in these vulnerable subsets. K) Radar summaries of communication strength among NR4A3_CMs, RGCC_CapECs, M1, and M2 macrophages (inferred by CellChat). MIR shifts hubs toward M1‐injury signaling; CSAδ re‐centers networks toward M2‐repair nodes. L) Ligand‐receptor dot plots (mean scaled expression; dot size, interaction probability; color, –log10 p) contrasting inflammatory axes (IL1B–IL1R1, TNF–TNFRSF1A, CCL2–CCR2) with reparative/angiogenic axes (VEGF–KDR, FGF2–FGFR1, ANGPT1–TEK, MFGE8–ITGAV/ITGB5, GAS6–MERTK, IGF1–IGF1R, FN1–Integrins). Data are mean ± SD (*n* = 3). one‐ or two‐way ANOVA with Tukey's post‐hoc: ^*^ *p*<0.05, ^**^ *p*<0.01, ^***^ *p*<0.005, ^****^ *p*<0.001. Abbreviations: snRNA‐seq, single‐nucleus RNA sequencing; UMAP, Uniform Manifold Approximation and Projection; CMs, cardiomyocytes; ECs, endothelial cells; capECs, capillary ECs; KEGG, Kyoto Encyclopedia of Genes and Genomes; GSEA, gene set enrichment analysis.

This study performed snRNA‐seq on infarct/border myocardium from aged Bama miniature pigs 3 days after MIR, with Sham, Saline, and CSAδ groups. After standard QC/integration, we obtained > 2 × 10⁴ high‐quality nuclei spanning major lineages (CMs, ECs, fibroblasts, mononuclear phagocytes; Figure S10A, Supporting Information). UMAP analyses highlighted two vulnerable subpopulations that were depleted by MIR and preserved by CSAδ: NR4A3⁺ cardiomyocytes (NR4A3_CMs) and RGCC⁺ capillary endothelial cells (RGCC_CapECs) (**Figure** **7**A). Their proportions fell sharply in Saline but rebounded with CSAδ when calculated against total cells and lineage baselines (Figure 7B–F). Pseudotime mapping placed both subtypes predominantly in early–intermediate states rather than terminally mature branches, indicating that MIR preferentially eliminates supply precursor‐like nodes, whereas CSAδ maintains lineage continuity (Figure S10C,D, Supporting Information). Pathway analysis linked the loss of these cells to cuproptosis. KEGG terms were enriched for cuproptosis, redox, and inflammatory pathways in Saline versus Sham (Figure 7G,H), and GSEA showed a positive association of cuproptosis with MIR and a negative association after CSAδ in both NR4A3_CMs and RGCC_CapECs (Figure 7I,J). Mononuclear phagocyte trajectories revealed MIR‐driven differentiation toward M1 macrophages with an early‐state M1 arm; CSAδ diverted trajectories toward M2 macrophages (Figure S10B, Supporting Information). Cell–cell communication centered on these four nodes (NR4A3_CMs, RGCC_CapECs, M1, M2) further clarified the microenvironmental shift: in Saline, signaling was dominated by inflammatory axes (IL1B‐IL1R1, TNF‐TNFRSF1A, CCL2‐CCR2), whereas CSAδ strengthened angiogenic axes (VEGF‐KDR, FGF2‐FGFR1, ANGPT1‐TEK, MFGE8‐ITGAV/ITGB5, GAS6‐MERTK, IGF1‐IGF1R, FN1‐Integrins) (Figure 7K,L). Together, the single‐nucleus data identify NR4A3⁺CMs and RGCC⁺capECs as early casualty populations in aged MIR, link their depletion to cuproptosis, and show that CSAδ preserves these nodes while reprogramming immune‐vascular communication toward repair.

### 2.8 CSAδ Disrupts CBX7–ATP7A LLPS to Suppresses Cuproptosis in Aged Pigs MIR Model

**Figure 8:**
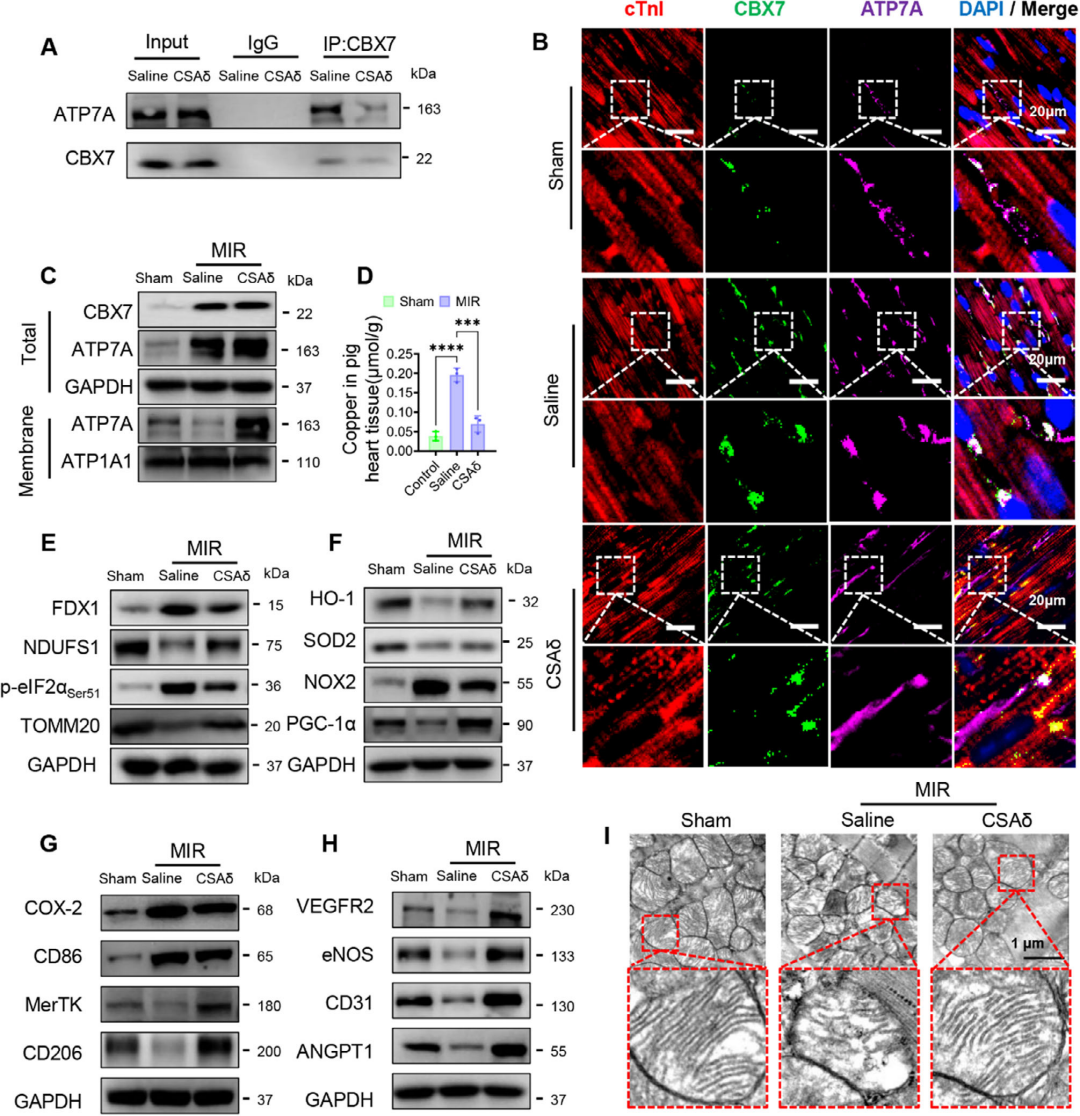
CSAδ disrupts CBX7‐ATP7A LLPS and restores ATP7A‐mediated copper efflux in aged pig. A) Co‐IP from infarct/border myocardium (24 h post‐MIR): ATP7A co‐precipitates with CBX7; the interaction is enhanced by MIR (Saline) and reduced by CSAδ. B) IF for cTnI (red), CBX7 (green), ATP7A (purple), DAPI (blue): MIR induces punctate CBX7‐ATP7A condensates in cardiomyocytes; CSAδ diminishes condensates and promotes membrane‐ward redistribution of ATP7A. Scale bars, 20 µm. C) WB of total versus membrane fractions: total CBX7 and ATP7A, and ATP7A in membranes (ATP1A1 control). CSAδ increases membrane ATP7A while total ATP7A remains relatively stable. D) Myocardial copper content (µmol g^−1^ tissue) in infarct region: MIR elevates copper versus Sham; CSAδ lowers copper versus Saline. E) Cuproptosis and mitochondrial proteins: MIR raises FDX1 and p‐eIF2α_Ser51_ and lowers TOMM20 and NDUFS1; CSAδ reverses these changes. F) Redox proteins: CSAδ decreases NOX2 and increases HO‐1, SOD2, and PGC‐1α, indicating reduced oxidative stress and improved mitochondrial biogenesis. G) Immune‐polarization markers: CSAδ lowers COX‐2 and CD86 (M1 and inflammatory) and elevates MerTK and CD206 (M2 and repair). H) Angiogenic and endothelial markers: CSAδ increases VEGFR2, eNOS, CD31, ANGPT1, consistent with pro‐angiogenic remodeling. I) TEM of cardiomyocyte mitochondria: Saline shows disorganized cristae and outer‐membrane disruption; CSAδ shows preserved cristae and intact outer membrane. Data are mean ± SD; one‐way ANOVA with Tukey's post hoc, ^***^ *p*<0.005, ^****^ *p*<0.001; ns, not significant. Abbreviations: MIR, myocardial ischemia/reperfusion; LLPS, liquid–liquid phase separation; Co‐IP, co‐immunoprecipitation; IF, immunofluorescence; WB, western blot; TEM, transmission electron microscopy; ATP1A1, Na⁺/K⁺‐ATPase α1; FDX1, ferredoxin‐1; eIF2α, eukaryotic initiation factor 2α; TOMM20, translocase of outer mitochondrial membrane 20; NDUFS1, NADH dehydrogenase subunit S1; HO‐1, heme oxygenase‐1; SOD2, mitochondrial superoxide dismutase; PGC‐1α, peroxisome proliferator‐activated receptor‐γ coactivator 1α.

In the aged pig MIR model, co‐immunoprecipitation showed that MIR strengthens the interaction between CBX7 and ATP7A, whereas CSAδ weakens this binding (**Figure** **8**A). Immunofluorescence (cTnI/CBX7/ATP7A/DAPI) revealed LLPS‐like CBX7‐ATP7A puncta in cardiomyocytes after MIR, which diminished with CSAδ; concomitantly, ATP7A redistributed toward the cytomembrane (Figure 8B). Fractionation western blots confirmed increased membrane ATP7A (ATP1A1 control) without major change in total ATP7A, and tissue copper in the infarct region fell with CSAδ (Figure 8C,D), indicating restored copper efflux. At the mitochondria‐cuproptosis axis, CSAδ reduced FDX1 and p‐eIF2α_Ser51_, while TOMM20 and NDUFS1 rebounded (Figure 8E). Oxidative‐stress readouts improved: NOX2 decreased, HO‐1, SOD2, and PGC‐1α increased (Figure 8F). Consistent with immune and vascular remodeling seen by single‐nucleus profiling, protein analyses showed COX‐2 and CD86 down, MerTK and CD206 up, and higher VEGFR2, eNOS, CD31, and ANGPT1 (Figure 8G,H). TEM illustrated preserved cristae and outer‐membrane continuity with CSAδ (Figure 8I). At the tissue level, CSAδ raised border‐zone pO_2_, lowered ROS/oxidative damage (DHE, 4‐HNE, 8‐oxo‐dG), increased the M2/M1 ratio at 1 and 3 days, and boosted CD31⁺ microvessels (Figure S11A–G, Supporting Information). Together with the cellular data, these results support a mechanism in which δAe within CSAδ inhibits CBX7, disrupting CBX7‐ATP7A LLPS, enabling ATP7A membrane translocation, enhancing copper efflux, and thereby suppressing cuproptosis while concurrently correcting redox, immune, and vascular imbalances in aged pig MIR.

## 3 Discussion

This study identifies CBX7 as an age‐contingent regulator of post‐ischemic repair that couples chromatin control to copper homeostasis in the heart. In young myocardium, CBX7 blockade facilitates cardiomyocyte cell‐cycle re‐entry; in aged myocardium, CBX7 instead engages ATP7A in LLPS condensates, sequestering ATP7A away from the membrane, reducing copper efflux, and licensing cuproptosis. Disrupting this condensate, genetically or with δ‐Amyrenone (δAe), restores ATP7A trafficking, lowers tissue copper, and suppresses mitochondrial stress, yielding structural, functional, and electrophysiologic benefit from cells to the whole heart. These findings extend the cuproptosis paradigm, copper binding to lipoylated TCA enzymes that triggers proteotoxic stress, to the aged heart and place ATP7A trafficking upstream of this death program.^[^ ^14^, ^22^ ^]^ Our results fit a broader theme of age‐dependent role switching in growth stress pathways. While classical effectors promote proliferation and anabolic programs in youth, their dominant outputs in aging shift toward stress resistance, metabolic control, and survival rather than cell division. Contemporary syntheses emphasize this pivot and provide a conceptual scaffold for why CBX7's most consequential action in the aged myocardium is to stabilize homeostasis rather than drive proliferation.^[^ ^23^, ^24^ ^]^ Mechanistically, this study shows that membrane‐associated condensates can tune transporter localization. LLPS is now recognized as a druggable, reversible state that organizes signaling and trafficking at membranes; in our setting, CBX7‐ATP7A droplets appear to retain ATP7A intracellularly when copper load is high, blunting the normal Golgi‐to‐plasma‐membrane shuttling required for efflux. δAe dissolves these droplets and rescues ATP7A membrane translocation, consistent with modern views of condensates as regulators of membrane dynamics and cargo routing.^[^ ^25^ ^]^ Equally important, this study connects this molecular axis to the injury niche using a materials strategy. The CSAδ hydrogel delivers δAe locally while correcting multiple hostile features of the post‐MIR microenvironment‐controlled oxygen release (CaO_2_), broad‐spectrum ROS buffering (melanin‐like nanoparticles, MNPs), pH stabilization (ascorbate), immune re‐programming toward M2, and electrical matching. Oxygen‐releasing and ROS‐scavenging biomaterials have advanced rapidly but often act along single pathways; by combining timed oxygenation with fast ROS interception and δAe's upstream control of copper‐mitochondrial stress, CSAδ addresses hypoxia, oxidative damage, immune skewing, and conduction heterogeneity in one injectable platform.^[^ ^26^, ^27^, ^28^ ^]^ Notably, MNPs are not redundant with δAe: MNPs provide early, multi‐species ROS quenching (DCFH‐DA/MitoSOX/Amplex Red kinetics), whereas δAe reduces ROS generation at the source by restoring ATP7A‐dependent copper efflux and mitochondrial proteostasis, yielding temporal and chemical complementarity. These molecular‐materials synergies translated from aged mice to aged Bama pigs, a large‐animal system with human‐like anatomy, coronary physiology, and arrhythmic susceptibility.^[^ ^29^, ^30^ ^]^ Single‐nucleus profiling pinpointed NR4A3⁺ cardiomyocytes and RGCC⁺ capillary ECs as vulnerable, early‐state subpopulations depleted by MIR yet preserved by CSAδ; pathway analysis linked their loss to cuproptosis, and communication analysis showed a shift from M1‐dominant inflammatory axes (IL1B‐IL1R1, TNF‐TNFRSF1A, CCL2‐CCR2) to repair/angiogenic axes (VEGF‐KDR, ANGPT1‐TEK, GAS6‐MERTK, IGF1‐IGF1R) after treatment, aligning with contemporary macrophage biology in cardiac repair. Together, these data establish a causal chain from δAe‐mediated condensate dissolution and ATP7A rescue to improved copper handling, mitochondrial integrity, microenvironment repair, and organ‐level function in aged hearts.

## 4 Conclusion 

This study defines an age‐targeted, mechanism‐anchored strategy for myocardial repair. In aged myocardium, CBX7 forms LLPS condensates with ATP7A that impede copper efflux and enable cuproptosis; δAe dissolves these condensates, restores ATP7A trafficking, and protects vulnerable NR4A3⁺ cardiomyocytes and RGCC⁺ capillary ECs. Embedding δAe in CSAδ adds spatiotemporally controlled delivery and microenvironment repair (oxygenation, ROS control, immune re‐programming, electrical matching), producing smaller infarcts, better pump function, and stabilized conduction in aged mice and pigs. Conceptually, the CBX7‐ATP7A‐copper axis emerges as a drug‐materials convergence point for elderly MIR, offering a template for precision cardiac therapeutics that respects age‐dependent protein plasticity while repairing the tissue niche.

### 4.1 Limitations and Future Directions

Although CSA and CSAδ were effective and well tolerated in mouse and aged pig models of myocardial ischemia–reperfusion, several hurdles remain for translation: manufacturing and quality control must be strengthened, including tight control of nanoparticle size and surface chemistry, validated sterilization, and stable in‐use windows despite ascorbic acid oxidation and calcium peroxide decomposition; delivery should be compatible with catheter or thoracoscopic procedures with a predictable gelation and adhesion window on a beating, bleeding surface, ideally via a ready‐to‐use metered applicator; long‐term safety requires definition of the oxygen‐release dose range, mitigation of pH and microbubble risks, surveillance for chronic immune activation, fibrosis, microcalcification, and electrophysiologic effects, and confirmation of melanin nanoparticle and degradation‐product clearance with feasibility of repeat dosing; study designs should account for patient and therapy heterogeneity, including disease stage, common comorbidities, antithrombotic regimens, and percutaneous coronary intervention; finally, as a drug‐device combination, the program must align with regulatory quality standards, clinically meaningful endpoints, and cost and access considerations.

## 5 Materials and Methods

### Establishment of Myocardial Ischemia‐Reperfusion (MIR) Model

Cellular Model. Primary cardiomyocytes were isolated from young adult (3–6 months) and aged (24 months) C57BL/6 mice to simulate myocardial infarction‐induced cellular injury. Cells were subjected to hypoxia‐induced injury by replacing the standard culture medium with glucose‐ and serum‐free DMEM and incubating them in a tri‐gas chamber (0.5% O_2_, 5% CO_2_) for 1 h to simulate ischemia (IF). Subsequent reperfusion was achieved through medium replacement with glucose‐containing DMEM under normoxic conditions (21% O_2_) for 24 h. Experimental animals included: Specific pathogen‐free (SPF) wild‐type C57BL/6 mice (WT, aged 2 years); Bama miniature pigs (WT, 15–20 kg); Cardiomyocyte‐specific CBX7 knockout mice (CBX7‐cKO, aged 2 years).

Murine Surgical Protocol. Anesthesia was induced via intraperitoneal injection of sodium pentobarbital (50 mg kg^−1^) followed by tracheal intubation connected to a rodent ventilator. Myocardial ischemia was monitored in real‐time using electrocardiography (ECG). A left thoracotomy was performed between the second and third intercostal spaces to expose the heart. Transient ischemia was induced by ligating the left anterior descending coronary artery (1 mm distal to its origin) with an 8–0 silk suture. Successful ischemia was confirmed by ST‐segment elevation and T‐wave inversion on ECG. After 45 min of occlusion, reperfusion was initiated by suture release.

Porcine Surgical Protocol. Following 12 h preoperative fasting, anesthesia was induced and maintained with isoflurane (1–2% inhalation) under tracheal intubation. Vital parameters (heart rate, respiratory frequency, SpO_2_) were continuously monitored. After thoracic hair removal and povidone‐iodine antisepsis, a median sternotomy with third intercostal extension was performed to access the heart. The second diagonal branch of the left coronary artery was occluded for 45 min followed by reperfusion. Postoperative closure used absorbable sutures in layers, with prophylactic antibiotics (cefazolin, 25 mg kg^−1^) and analgesia (lidocaine, 2 mg kg^−1^). Successful myocardial infarction was reconfirmed by intraoperative ECG. All subjects were euthanized via sodium pentobarbital overdose (150 mg kg^−1^) followed by necropsy for organ collection.

### Ethics Statement

All animal procedures complied with institutional and national guidelines and were approved by the Animal Ethics Committee of Sichuan Provincial People's Hospital (Approval No.: 2024‐08). Animal housing and procedures followed the ARRIVE guidelines and institutional SOPs, with efforts to minimize suffering and reduce animal use.

### Copper Content Quantification

Cells were harvested and transferred into centrifuge tubes. After centrifugation (300 × *g*, 5 min), the supernatant was discarded and pellets were resuspended in distilled water (0.4 mL per 1 × 10⁶ cells). Cell suspensions were sonicated on ice using a probe‐type ultrasonic homogenizer (200 W output, cycles of 3 s pulses alternating with 7 s intervals for a cumulative 3 min). The resultant lysates were centrifuged at 10 000 × *g* for 10 min at 4 °C. Supernatants were collected for copper quantification using a commercial assay kit (BC5755; Solarbio, Beijing, China) according to the manufacturer's protocol.

Under deep anesthesia, PBS‐perfuse via LV to clear blood, excise LV, blot, and sample infarct/border/remote regions. Weigh 20–30 mg wet tissue, add metal‐free distilled water (10 µL mg^−1^), homogenize on ice (ceramic/plastic tools), clarify 10 000 × g 10 min at 4 °C, and assay supernatant with BC5755 in duplicate alongside standards/blanks. Report µmol g^−1^ wet tissue using the kit concentration and extraction volume; optionally normalize to protein (BCA) as µmol mg^−1^ protein. Minimize metal contamination and keep extracts cold; store at −80 °C if needed.

### Single‐Nucleus RNA‐seq (snRNA‐seq) and Computational Analysis

Nuclei were isolated from frozen left ventricular samples (Sham, Saline, CSAδ) following detergent/mechanical dissociation with sucrose cushion cleanup and DAPI gating. Libraries were prepared with a droplet‐based 3′ snRNA‐seq kit compatible with the CeleLens Cloud pipeline and sequenced to a depth of ≈50 000 reads/nucleus (paired‐end). Raw data were processed on CeleLens Cloud (https://www.celelenscloud.cn/) using its standardized workflow: i) read trimming and alignment to Sus scrofa reference (Sscrofa11.1) with intronic counting enabled; ii) cell/nucleus calling and UMI de‐duplication; iii) quality control (mitochondrial transcripts < 10–15%, detected genes/nucleus within MAD‐filtered bounds, doublet removal); iv) normalization and variance stabilization (SCTransform), feature selection, PCA, and batch correction (Harmony); v) graph construction, Louvain/Leiden clustering, and UMAP embedding; vi) differential expression (Wilcoxon rank‐sum, Bonferroni/Benjamini–Hochberg adjusted p) with thresholds |log2FC| > 0.25–0.5 and adjusted *p* < 0.05; vii) pathway enrichment by KEGG/GSEA; viii) pseudotime/trajectory inference for mononuclear phagocytes and endothelial subclusters (Monocle‐style lineage‐aware methods on the CeleLens platform); and ix) ligand–receptor analysis (CellPhoneDB‐/NicheNet‐like module on CeleLens). For cross‐species ligand–receptor mapping, porcine genes were mapped to human orthologs (Ensembl/biomaRt) before querying the human L–R atlas integrated in CeleLens Cloud. Detailed parameters and visualization settings (UMAP, violin, velocity/trajectory plots) followed CeleLens defaults unless otherwise specified.

### Synthesis of CSA Hydrogel

Under aseptic conditions (biosafety cabinet), an optimized one‐pot CSA hydrogel was prepared with the final composition of SA 2.0% w/v, calcium gluconate (CaGlu) 80 mm, calcium peroxide (CaO_2_) 0.12 mg mL^−1^, melanin nanoparticles (MNPs) 0.15 mg mL^−1^, and ascorbic acid (AsA) 2 mm at pH 7.2–7.4 using a B:A = 3:1 (v/v) mixing scheme. Component A was prepared by dissolving calcium gluconate to 320 mm in sterile water (37–50 °C with gentle stirring) and dispersing CaO_2_ nanoparticles to 0.48 mg mL^−1^ by low‐shear homogenization with brief bath sonication, followed by 3–5 min vacuum degassing and light protection. Component B was prepared by dissolving SA to 2.67% (w/v) at 37 °C, adding MNPs to 0.20 mg mL^−1^ and AsA to 2.67 mm, mixing for 10–15 min, and degassing (vacuum 5–10 min or 500–800 g for 3 min). For one‐pot mixing, Component A was added dropwise into B under 800–1200 rpm stirring over 1–2 min, followed by 10 min additional stirring to promote Ca^2^⁺ diffusion and early network formation; the mixture was then degassed again (500–800 g, 3 min, or vacuum 5 min) to yield a uniform injectable pre‐gel, equilibrated at 37 °C and used within ≤2 h. After mixing, the system reaches the target final concentrations; all steps were performed with light protection, storing A (CaO_2_‐containing) and B (AsA‐containing) separately until use.

Synthesis of CSAδ Hydrogel: The preparation of CSAδ hydrogel was similar to that of the CSA hydrogel, with the only difference being the addition of δAe to Component B prior to mixing with Component A. The δAe was added to achieve a final gel concentration of 0.5 mg mL^−1^, after which the synthesis continued following the same steps as for the CSA hydrogel.

### Characterization

The chemical structures of the SA and CSA hydrogels were characterized by Fourier‐transform infrared spectroscopy (FT‐IR), and their rheological properties were analyzed using a rheometer.

### Statistical Analysis

All quantitative data are expressed as mean ± standard deviation (SD). Statistical analyses were performed using GraphPad Prism. An unpaired two‐tailed Student's *t*‐test was used for comparisons between two groups. For comparisons among multiple groups with a single factor, one‐way ANOVA followed by Tukey's post hoc test was used. For comparisons involving two independent variables, two‐way ANOVA with appropriate post hoc tests was applied. A *p*‐value < 0.05 was considered statistically significant.

Additional materials and methods are supported in the Supporting Information.

## Conflict of Interest

The authors declare no conflict of interest.

## Supporting information



Supporting Information

## Data Availability

The data that support the findings of this study are available on request from the corresponding author. The data are not publicly available due to privacy or ethical restrictions.
