# Supplementary material for: Engineered Multifunctional Hydrogel Delivering Novel CBX7 Inhibitor Modulates Cuproptosis Via Liquid–Liquid Phase Separation to Restore Cardiac Function in Aged Myocardial Infarction
Source: Adv Sci (Weinh). 2025 Oct 21;13(1):e11630. doi: 10.1002/advs.202511630 (PMC12767114; doi:10.1002/advs.202511630)
Supplement: Supplementary file 1 — Supporting Information [file ADVS-13-e11630-s001.docx]

**Support information**

**Engineered Multifunctional Hydrogel Delivering Novel CBX7 Inhibitor Modulates Cuproptosis via Liquid–Liquid Phase Separation to Restore Cardiac Function in Aged Myocardial Infarction**

Jun Liu^1,2,3^†, Peng Qu^1,2,3^†, Jiao Shi^1,2,3^†, Tingting Liang^4^, Yao Gu^1,2,3^, Xue Li^1,2,3^, Cui Ma^5^, Danyang Zhao^6^, Feila Liu^7^, Qi Liang^1,2,3^*, Panke Cheng^4,8^*, Qian Lei^6^*

1. Department of Clinical Laboratory, Affiliated Hospital of North Sichuan Medical College, Nanchong, 637000, China.

2. School of Laboratory Medicine, North Sichuan Medical College, Nanchong, 637007, China.

3. Translational Medicine Research Center, North Sichuan Medical College, Nanchong, 637007, China.

4. Institute of Cardiovascular Diseases & Department of Cardiology, Sichuan Provincial People's Hospital, School of Medicine, University of Electronic Science and Technology of China, Chengdu, 610072, China

5. Department of Mathematics, Army Medical University, Chongqing, 400038, China.

6. Department of Anesthesiology, Sichuan Provincial People's Hospital, School of Medicine, University of Electronic Science and Technology of China, Chengdu 610072, China

7. School of Pharmacy and Bioengineering, Chongqing University of Technology, Chongqing 400050, China

8. Ultrasound in Cardiac Electrophysiology and Biomechanics Key Laboratory of Sichuan Province, Chengdu, 610072, China

†These authors contributed equally.

* ***Correspondence to:* Qian Lei** (E-mail: [leiqianggh@163.com](mailto:leiqianggh@163.com)), **Panke Cheng** (E-mail: [chengpk1002@163.com](mailto:chengpk1002@163.com)) and **Qi Liang** (Email: liangqineijiang@126.com).

**Supplementary Materials and Methods**

***Western Blot Analysis & Co-immunoprecipitation (Co-IP)***

Tissue/cellular lysates were prepared using RIPA buffer (R0010; Solarbio, China) supplemented with protease/phosphatase inhibitors. Protein concentration was determined via BCA assay (PC0020; Solarbio). Equal amounts (30 μg/lane) of proteins were separated on 10% SDS-PAGE gels and transferred to PVDF membranes (0.22 μm pore size; Millipore). Membranes were blocked with 5% non-fat milk in TBST (Tris-buffered saline with 0.1% Tween-20) for 1 h at room temperature, followed by incubation with primary antibodies (4°C, 12 h) and HRP-conjugated secondary antibodies (37°C, 1 h). Signals were developed using ECL Prime (GE Healthcare) and quantified via Amersham ImageQuant 800 system (Cytiva, China). Primary antibodies from Abcam: CBX7 (ab21873), GAPDH (ab8245), Cyclin D1 (ab134175), Cyclin A2 (ab32386), CDK2 (ab32147), ALDH7A1 (ab53278), SIRT7 (ab259968), FOXM1 (ab207298), ATP7A (ab308524), ATP1A1 (ab300507), VWF (ab6994), VCAM-1 (ab134074), CD31 (ab9498), FDX1(ab108257), p-eIF2α_Ser51_(ab32157), TOMM20 (ab186735), NDUFS1 (ab169540), NOX2 (ab310337), HO-1 (ab189491), SOD2 (ab68155), PGC-1α(ab191838), COX-2 (ab179800), CD86 (ab220188), MerTK (ab300136), CD206 (ab64693), VEGFR2 (ab315238), eNOS (ab317582), CD31 (ab281583), ANGPT1 (ab133425).

Protein complexes were isolated using Immunoprecipitation Kit (Protein A/G Agarose Beads Method, P2197S; Beyotime). Briefly: Pre-clearance: 500 μg lysate incubated with control beads (1 h, 4°C).Antibody coupling: 2 μg primary antibody bound to Protein A/G beads (30 min, RT).Target capture: Antibody-bead complexes incubated with pre-cleared lysates (overnight, 4°C).Washes: 5× with IP buffer (25 mM Tris-HCl pH7.5, 150 mM NaCl, 0.5% Triton X-100).Elution: Boiling in 2× Laemmli buffer (95°C, 5 min).Co-precipitated proteins were analyzed by Western blot using reciprocal antibodies.

***Detection of cTnI and CK-MB***

The levels of cardiac troponin I (cTnI) were measured using species-specific ELISA kits. For pigs, the Porcine TNNI3 ELISA Kit (ELK5656; ELK Biotechnology, China) was employed, while for mice, the Mouse cTnI ELISA Kit (D721149; Sangon Biotech, China) was used. All procedures were conducted according to the manufacturers’ instructions. Similarly, creatine kinase-MB (CK-MB) levels were quantified using the corresponding ELISA kits for each species: the Porcine CK-MB Kit (CB10105-Pg; Coibo, China) for pigs, and the Mouse CK-MB Kit (SEKM-0152; Solarbio, China) for mice. Following the successful establishment of each experimental model, 0.2 mL of venous blood was collected for the assays.

***Bioinformatic Analysis***

RNA sequencing was performed by Tsingke Biotechnology Co., Ltd. (Beijing, China) using myocardial cells from young and aged mice treated with IF+DMSO or IF+δ-Amyrenone(δAe). Raw sequencing data were processed through the TSINGKE Cloud Platform (<https://cloud.tsingke.com.cn/>) with built-in analytical pipelines. Differential expression analysis was conducted using the platform's DESeq2-based algorithm. Genes with absolute log₂ fold change (|log₂FC|) > 1 and Benjamini-Hochberg adjusted *P* < 0.05 were identified as statistically significant, visualized through volcano plots generated by ggplot2 package (v3.4.2). Pathway enrichment analysis was performed through gene set enrichment analysis (GSEA) using Kyoto Encyclopedia of Genes and Genomes (KEGG) database (2023 release). Significant pathways were determined by normalized enrichment score (NES) > 1.5 and false discovery rate (FDR) < 0.25. All analytical workflows strictly followed the platform's standardized protocols to ensure reproducibility.

***Cell Viability and Death Analysis***

Cellular viability was assessed using the Cell Counting Kit-8 (CCK-8, C0037; Beyotime Biotechnology, China) following the manufacturer's protocol. Apoptotic and necrotic populations were discriminated through dual-staining strategies: YO-PRO-1 Assay: Cells were incubated with 5 μM YO-PRO-1 (C2022; Beyotime) for 30 min at 37°C in darkness, with fluorescence imaging performed using an inverted fluorescence microscope. Annexin V/PI Assay: Cells were stained with Annexin V-FITC (C1062M; Beyotime) and propidium iodide (PI, 2 μg/mL) in binding buffer for 15 min prior to flow cytometric analysis (BD FACS Canto II). Quantitative data were processed using FlowJo software (v10.8.1).

***Mitochondrial Reactive Oxygen Species (mtROS) Detection***

Mitochondrial superoxide was detected with MitoSOX™ Red (M36005; Invitrogen, USA) with mitochondrial counterstaining by MitoTracker Deep Red FM (C1032; Beyotime, China). A 10 µM MitoSOX working solution was prepared by diluting 1 µL of 5 mM stock in 0.5 mL PBS; MitoTracker was reconstituted to 200 mM in anhydrous DMSO (50 µg in 460 µL) and diluted 1:2,000 in PBS to 40 nM. After aspirating the culture medium and one PBS rinse, cells were incubated in the dark with PBS containing MitoSOX 10 µM and MitoTracker 40 nM at 37 °C, 5% CO₂ for 30 min, washed twice with PBS, and imaged immediately in fresh PBS on a confocal microscope using fixed acquisition settings across groups.

***Immunoprecipitation Mass Spectrometry (IP-MS)***

Primary cardiomyocyte lysates were prepared in ice-cold RIPA buffer (P0013B; Beyotime Biotechnology, China) supplemented with protease inhibitor cocktail (1:100). After centrifugation (12,000 × *g*, 10 min, 4°C), protein concentration was determined via BCA assay (PC0020; Solarbio, China). 1 mg total protein was incubated with anti-CBX7 antibody (10 μg, ab21873; Abcam, USA) pre-conjugated to Protein A/G magnetic beads (P2197S; Beyotime) through rotation (12 rpm) at 4°C for 16 h. Parallel control experiments used normal rabbit IgG (2729S; Cell Signaling Technology). Beads were subsequently washed five times with high-stringency buffer (50 mM Tris-HCl pH 7.4, 150 mM NaCl, 0.5% NP-40). Bound proteins were eluted using 2× Laemmli buffer (70°C, 10 min). Eluates were reduced with 10 mM DTT (30 min, 56°C), alkylated with 55 mM iodoacetamide (30 min, dark), and digested with sequencing-grade trypsin (1:50 w/w, 37°C, 16 h). Peptides were desalted using C18 StageTips prior to LC-MS/MS analysis. Samples were analyzed on an Orbitrap Exploris 480 mass spectrometer (Thermo Scientific) coupled to an EASY-nLC 1200 system. Chromatographic separation used 75 μm × 25 cm PepMap column (2 μm C18) with 120-min gradient (5-35% acetonitrile/0.1% formic acid). Raw files were processed through MaxQuant (v1.6.17.0) against UniProt Mouse database.

***Real-time fluorescence quantitative PCR***

RNA Isolation:Total RNA was extracted using SteadyPure Tissue/ Cell Small RNA Kit(AG21023; Accurate Biology, China)under RNase-free conditions. RNA purity was verified by Nanodrop (A260/A280:1.9-2.1). cDNA Synthesis:Genomic DNA elimination and reverse transcription were performed using Evo M-MLV RT Kit (AG11728; Accurate Biology) with 1 μg total RNA. qPCR Amplification:Real-time fluorescent quantitative PCR amplification of target genes was performed using SYBR Green Pro Taq HS premixed qPCR kit (AG11718, Ecoray, China). Data Analysis: Relative quantification was calculated by 2^(-ΔΔCt) method. *CBX7* (Forward: 5′-GAAGAGGAGGCAGAAGCAGACC-3′, Reverse: 5′-TGACGGAGTTGGCGGTGATG-3′), *GAPDH (*Forward: 5′-GGTGGACCTCATGGCCTACA-3′, Reverse: 5′-CTCTCTTGCTCTCAGTATCCTTGCT-3′).

***Immunofluorescence Staining***

Cellular Staining Protocol: Fixation: Cells were fixed with 4% paraformaldehyde (PFA) for 15 min at RT. Permeabilization: Treated with 0.5% Triton X-100 in PBS (10 min, RT). Blocking: Incubated with 5% goat serum for 1 hour. Application of primary antibody: Overnight incubation at 4°C with primary antibody. Secondary Antibodies: Alexa Fluor-conjugated antibodies (Invitrogen) for 1 hour (dark). Nuclear Counterstain: DAPI (Sigma) for 5 min. Mounting: Coverslipped after dropwise application of antifade mounting medium. Imaging: Acquired on a fluorescence microscope.

Tissue Staining Protocol: Fixation: Fresh tissues were immersion-fixed in 4% PFA for 24 h at 4°C.Processing: Dehydrated through ethanol gradients, xylene-cleared, and paraffin-embedded. Sectioning: 5 μm sections mounted on slides. Deparaffinization: Xylene (3×10 min) to 100%-70% ethanol series. Antigen Retrieval: Heated in citrate buffer (pH 6.0) at 95°C for 20 min. Other steps are the same as cellular immunofluorescence. Primary Antibodies: CBX7 (TD13257; Abmart ;China), KI67 (A26419PM;abclonal;China), CTNI (66376-1-Ig; Proteintech, China), CD86 (13395-1-AP; Proteintech; China), CD206 (18704-1-AP; Proteintech; China),CD31 (A19014; Abclonal; China).

***Surface Plasmon Resonance (SPR) Analysis***

Affinity measurements were performed using the Octet label-free molecular interaction analysis system (Sartorius, Germany). In the experiment, biotinylated CBX7 protein was directly immobilized on the surface of SAA biosensors. Prior to immobilization, the SAA sensors were activated in PBS buffer for 10 minutes. The small-molecule compound δAe was serially diluted in running buffer to concentrations of 100, 50, 25, 12.5, 6.25, and 3.1 μM, and injected into the sample channels for analysis. Each concentration of δAe was injected into the Fc2-Fc1 channel at a flow rate of 30 μL/min, with an association time of 60 seconds and a dissociation time of 90 seconds. Each detection cycle consisted of sample injection, molecular binding, and dissociation phases, all conducted under identical running buffer conditions. Signal changes during the baseline, association, and dissociation phases were recorded to analyze the binding interactions between the small molecule and the target protein. Experimental data were processed and fitted using the Octet Data Analysis Software to evaluate affinity parameters between the interacting molecules.

***Preparation and Characterization of Melanin Nanoparticles (MNPs), CaO_2_ NPs.***

Preparation of MNPs:An appropriate amount of ink sac was extracted and added to distilled water. The mixture was thoroughly mashed and stirred, then uniformly agitated on a magnetic stirrer for 12 hours to dissolve lipids and other impurities. Afterward, the suspension was allowed to settle so that the precipitate could form, and the supernatant was discarded. Distilled water was added again to the precipitate, and the washing step was repeated a total of six times. Finally, the washed precipitate was freeze-dried to obtain the melanin nanoparticles.

Preparation of Calcium Peroxide Nanoparticles (CaO₂ NPs): Three grams of calcium chloride were dissolved in 30 mL of distilled water. Subsequently, 15 mL of 1 M ammonia and 120 mL of polyethylene glycol 200 were added, and the solution was stirred thoroughly at room temperature until homogeneously mixed. Then, 30 mL of 30% H₂O₂ solution was slowly added dropwise to the mixture, with each drop being added at an interval of 20–30 seconds. Once the H₂O₂ addition was complete, ammonia was further added dropwise to adjust the pH of the solution to 10, during which a white or pale-yellow precipitate gradually formed. The reaction was allowed to proceed with continuous stirring for an additional 2 hours to ensure complete reaction. The resulting reaction mixture was then centrifuged at 12,000 g for 5 minutes to separate the precipitate, and the supernatant was discarded. The precipitate was washed three times with a NaOH solution at pH 13 and subsequently washed twice with distilled water. Finally, the washed precipitate was placed in a vacuum oven at 80°C and dried for 2 hours to yield the final CaO₂ nanoparticles.

Characterization: Fourier-transform infrared spectroscopy (FT-IR) was employed to characterize the chemical structures of both MNPs and CaO₂ NPs. The crystal structure of CaO₂ NPs was analyzed by X-ray diffraction (XRD). In addition, the surface morphology and physical structure of MNPs and CaO₂ NPs were observed and characterized using scanning electron microscopy (SEM).

***Preparation and Characterization of Hydrogel***

Synthesis of SA Hydrogel: A 3% (w/v) solution of calcium gluconate and a 3% (w/v) solution of sodium alginate were first prepared. The sodium alginate solution was then mixed with the calcium gluconate solution at a volume ratio of 3:1. This mixture was continuously stirred for 10 minutes to promote the reaction. Finally, bubbles were removed by centrifugation, resulting in a homogeneous CSA hydrogel solution.

***CSA Hydrogel Formulation Optimization***

A two-stage, multi-objective strategy was used to optimize the CSA hydrogel formulation. Stage I employed a fractional factorial design with center-point replicates to screen five factors and their interactions. Factor levels were: sodium alginate (SA, % w/v): 1.2, 2.0, 2.8, 3.0, 3.5; calcium gluconate (CaGlu, mM): 40, 60, 80, 100, 120; calcium peroxide (CaO₂, mg/mL): 0.06, 0.12, 0.18, 0.25; melanin nanoparticles (MNPs, mg/mL): 0.08, 0.12, 0.15, 0.20, 0.25; and L-ascorbic acid (AsA, mM): 1, 2, 3, 5, 6. Primary objectives were wet adhesion/interfacial strength and in vivo retention, quantified by Tack σmax(kPa), Wad(J/m²), Lap shear τmax(kPa), Peel Gc(J/m²), and retention half-life T₅₀. Secondary targets were gel time 60-120 s, post-injection G′ at 10 rad/s of 3-6 kPa, tan δ < 0.5, 27 G plunger force ≤ 10 N, and pH drift ≤ 0.3. Safety/biocompatibility served as hard constraints (cardiomyocyte co-culture viability ≥ 80% at 24-72 h; hemolysis < 5%). Effect Pareto charts and ANOVA (P < 0.05) identified main effects and significant interactions; low-contribution or risky combinations were excluded (e.g., CaO₂ > 0.20 mg/mL due to pH excursions and reduced cell viability). Stage II focused on SA, CaGlu, CaO₂, and MNPs, fitting quadratic response surfaces with adequacy criteria of adjusted R² > 0.90 (for most endpoints), lack-of-fit P > 0.10, normal residuals, and VIF < 5. Multi-objective optimization used a desirability function D=(d_1_^W1^×d_2_^W2^×⋯×d_n_^Wn^)^1^/∑W with weights of 0.50 for the adhesion metrics (σmax, Wad​, τ_max_ and Gc), 0.30 for T₅₀, 0.15 for gel time/G′/injectability, and 0.05 for pH drift; noncompliant cytocompatibility runs were discarded. The analysis indicated that increasing MNPs and CaGlu most strongly enhanced wet adhesion via catechol-like chemistry and Ca²⁺-carboxylate ionic bridging, with a positive interaction; raising SA increased G′ and anti-washout but above 2.8% (w/v) led to higher plunger force, unfavorable tan δ, and excessive local stiffness; CaO₂ at 0.10-0.15 mg/mL⁻¹ improved interfacial densification and retention, whereas higher levels triggered pH elevation and cell stress. Balancing desirability and operability yielded the recommended formulation: SA 2% (w/v), CaGlu 80 mM, CaO₂ 0.12 mg/mL, MNPs 0.15 mg/mL, and AsA 2 mM.

***Rheology***

Oscillatory rheology was performed on a stress-controlled rheometer with 20 mm parallel plates, gap 1.0 mm, 37 °C. Pre-gel precursors were assessed by flow sweeps (0.1-100 s⁻¹) for shear-thinning; gels were tested after 30 min cure by frequency sweeps (0.1-100 rad/s, 1% strain within LVR) to obtain G′, G″, and tan δ. Each condition used n = 3 independent gel discs.

***Mercury intrusion porosimetry (MIP)***

Lyophilized prisms (5 × 5 × 2 mm) were loaded into calibrated penetrometers, vacuum-degassed (<50 mTorr), and run on an automated porosimeter using standard Hg constants (γ = 0.485 N/m; θ = 140°). Low-pressure and high-pressure stages covered 10 nm–100 µm equivalent throat diameters via the Washburn equation. Reported parameters: cumulative intrusion vs diameter, dV/dlogD, apparent porosity (% of bulk volume), modal throat size, and total intruded volume (mL/g). n = 3 independent gels/group.

***Adhesion to wet myocardium***

Fresh pig left-ventricular tissue was trimmed to flat coupons and kept hydrated at 37 °C in PBS. Gels were formed between one tissue face and a polycarbonate or stainless-steel counterface. Probe-tack: 5 mm probe; contact 10 kPa for 60 s; retract 100 µm/s. Readouts: peak stress (σ_max_) and work of adhesion (Wad). Lap-shear: Overlap 10 × 20 mm; crosshead speed 5 mm/min. Readout: τ_max_. 180° peel: Strip width 20 mm; rate 50 mm/min. Readout: peel energy (Gc) derived from force–distance curves. All tests at 37 °C; n = 5 bonds/assay/group. Substrates were randomized; operators were blinded to formulation labels.

***Stability and release***

Hydrogel degradation, oxygen release, δAe release, and pH change were assayed in parallel using identical discs (8 mm diameter, 2 mm thickness) prepared from SA, CSA, or CSAδ and incubated at 37 °C under sink conditions (25 mL medium/disc, gentle orbital agitation 60 rpm) in three media that model distinct environments: PBS + 1.8 mM CaCl_2_ (ionic crosslink–stabilizing), PBS + alginate lyase 1 U/mL (enzymatic degradation), and isotonic saline containing protein (0.9% NaCl + 1% FBS) (protein-rich milieu). Prior to immersion, each disc was blotted to remove surface moisture and weighed (m0); at predetermined time points, discs were removed, briefly blotted, weighed in the swollen state (msw), then dried to constant mass at 37 °C under vacuum and reweighed (mdry) to calculate swelling ratio (Q = msw/m0) and percentage mass remaining (mdry/m0 × 100%); separate discs were used for destructive time points to avoid handling artifacts (n=3 per group/condition). Dissolved O_2_ was recorded continuously through a septum using a calibrated fiber-optic oxygen probe inserted into the sealed vessel, with baseline medium O_2_ logged for 10 min before disc addition and with daily two-point probe calibration at 0%/air saturation; data were exported as mg/L versus time. Medium pH was measured in situ with a micro-pH electrode equilibrated at 37 °C, logging at the same time points as gravimetry; probes were rinsed between measurements and verified against pH 7.00/7.40 standards. For δAe release (CSAδ only), the supernatant was sampled at each time point (1.0 mL), immediately filtered (0.22 µm), protected from light, and quantified by HPLC-UV on a C18 column (4.6 × 150 mm, 5 µm; 30 °C; mobile phase acetonitrile/water 80/20 with 0.1% formic acid; flow 1.0 mL/min; detection 210 nm) using an external standard curve (0.5–50 µg/mL, r² ≥ 0.999); equal volumes of fresh, pre-warmed medium were returned after sampling to maintain sink conditions, and blank media and SA/CSA (no δAe) served as controls for background subtraction.

***In vivo retention (fluorescent labeling)***

For retention tracking, gels were labeled with rhodamine B (0.02 mg/mL in the pre-mix; protected from light) and injected intramyocardially immediately after mixing. Whole-organ fluorescence imaging was performed at predetermined time points under identical exposure. Regions of interest (ROIs) encompassing injection sites were quantified as radiant efficiency and normalized to the day-0 value to estimate retention half-life (T₅₀). FITC-labeled gels were used in separate animals for histological co-localization (anti-cTnI) to verify apposition and absence of gross cavities. All animal procedures followed the approved protocol and were randomized.

***Cardiomyocyte–macrophage co-culture for polarization (4:1)***

Cardiomyocyte–macrophage co-culture for polarization (4:1). Primary aged mouse cardiomyocytes and macrophages were co-cultured at a 4:1 (CM:Mac) ratio in collagen-coated 24-well plates. Cardiomyocytes were subjected to oxygen-glucose deprivation/reperfusion by incubation at 0.5% O₂ in glucose- and serum-free DMEM for 1 h, then returned to glucose-containing DMEM under normoxia for 24 h; during the reoxygenation phase, cultures were treated with SA or CSA hydrogels that had been pre-equilibrated in culture medium, with a vehicle control processed in parallel. At a total culture time of 72 h, macrophage phenotype was assessed by flow cytometry and immunofluorescence and cytokines were measured in supernatants. For flow cytometry, cells were detached with Accutase, passed through a 40 µm strainer, blocked with Fc buffer, stained with anti-CD86 (APC) and anti-CD206 (PE), and viability was assessed with propidium iodide; compensation used single-stain controls, and gating proceeded sequentially through FSC/SSC, singlets, live cells, and CD86/CD206 quadrants, acquiring at least 10,000 events per sample on a benchtop cytometer, with analysis in FlowJo v10.8. For immunofluorescence, cells on coverslips were fixed, permeabilized, incubated with antibodies against CD86 or CD206, counterstained with DAPI, and imaged under identical exposure settings; quantification used ImageJ with matched thresholds across groups. Supernatants were collected without disturbing the cell layer and analyzed by ELISA for IL-1β and TNF-α in duplicate wells.

***Redox assays***

Redox assays in aged primary mouse cardiomyocytes after OGD/R. Ventricular cardiomyocytes were isolated from 24-month-old C57BL/6 mice, plated on collagen-coated plates/coverslips (≥24 h to recover), and subjected to oxygen-glucose deprivation/reperfusion (OGD/R: 0.5% O₂ in glucose- and serum-free DMEM for 1 h, then normoxic reoxygenation in glucose-containing DMEM for 24 h); redox readouts were acquired during reoxygenation (unless otherwise noted), with identical acquisition settings across groups and biological n≥3. Total intracellular ROS was measured by loading cells with DCFH-DA 10 µM in warm HBSS for 30 min at 37 °C in the dark, followed by three brief washes in HBSS and immediate imaging on an epifluorescence microscope or reading on a plate reader (Ex/Em 485/535 nm); background (cell-free well) was subtracted and signals were normalized to cell number (DAPI nuclei counts) or total protein (BCA). Mitochondrial superoxide was assayed by incubating cells with MitoSOX Red 5–10 µM for 10 min at 37 °C together with MitoTracker Deep Red 40 nM for 15–30 min (sequential or co-incubation), washing in pre-warmed HBSS, and imaging by confocal microscopy using fixed laser power, gain, and pinhole; mitochondrial regions of interest were defined from the MitoTracker channel, and mean MitoSOX intensity per cell was quantified after rolling-ball background subtraction in ImageJ. Extracellular H₂O₂ was quantified kinetically using Amplex Red (50 µM) with horseradish peroxidase (0.1 U/mL) prepared in phenol-red-free HBSS; 100–200 µL reaction solution was added to conditioned medium, plates were maintained at 37 °C, and fluorescence (Ex/Em 560/590 nm) or absorbance (563 nm) was recorded every 3–5 min for 30–60 min. A freshly prepared H₂O₂ standard curve (0–5 µM) in the same matrix was run on each plate for calibration; catalase (500 U/mL) and no-cell wells served as negative controls. For kinetic analysis, per-well traces were baseline-corrected and fit to a single-exponential model to derive the apparent half-life (t₁/₂) and initial rate; technical duplicates were averaged before statistics. All reagents were equilibrated to 37 °C.

***Cell Cycle Analysis of Cardiomyocytes***

The effect of δAe on the cell cycle of cardiomyocytes from ischemia-reperfusion (young/adult and elderly) mice was evaluated using a cell cycle analysis kit (C1052, Beyotime, Beijing). After collecting the treated cardiomyocytes, staining was performed according to the kit manufacturer's instructions. Subsequently, flow cytometry was used to detect and analyze the distribution of cardiomyocytes across the different cell cycle phases (G1, S, and G2/M).

***Electrical Conductivity Testing***

The electrical conductivity of both SA and CSA hydrogels was evaluated using an LED circuit experiment. Additionally, their conductivities were measured using an electrochemical workstation (JG, ST2253y, China).

***Cell Viability Assay***

The effect of the hydrogel samples on the viability of primary cardiomyocytes derived from elderly mice was evaluated using the CCK-8 assay. In the experiment, hydrogels at different concentrations (0–20%) were co-cultured with the cells for 24, 72, and 168 hours, and their impact on cell viability was determined. Additionally, the effect of the hydrogels on the viability of human umbilical vein endothelial cells (HUVECs) under ROS-induced oxidative stress was also analyzed by the CCK-8 assay.

***Drug Injection and Hydrogel Implantation***

Mice: After reperfusion, experimental mice received an intramyocardial injection of 20 μL PBS. One group was injected with PBS without δAe, while the other group received PBS containing 0.025 mg δAe. For the hydrogel-treated groups, 20 μL of different types of hydrogels were injected into the infarcted and border zones of the myocardium. The thoracic cavity was then sutured layer by layer, ensuring that the intrathoracic gas was evacuated. A series of post-operative experiments were subsequently conducted to assess the efficacy of the various treatment regimens. Pigs: After reperfusion, pigs in the saline group received an intramyocardial injection of 0.5 mL of physiological saline, while those in the hydrogel-treated group were administered 0.5 mL of CSAδ hydrogel via multiple injection points into the infarcted and peripheral regions of the myocardium. The thoracic cavity was closed in layers and any intrathoracic gas was removed. A series of post-operative evaluations were then performed to compare the therapeutic effects of the different treatment protocols.

***Protein Purification***

The expression plasmid of each protein was constructed by Sangon biotech (China) and transformed into receptive Escherichia coli. The plasmids carried a kanamycin resistance gene; after transformation, the bacterial culture was plated on LB agar plates containing kanamycin and incubated at 37°C overnight to select positive colonies. A single positive colony was then inoculated into LB liquid medium with kanamycin and cultured overnight in a shaking incubator at 37°C. A small volume of this culture was subsequently transferred into fresh antibiotic-containing medium and grown until the OD₆₀₀ reached 0.6–0.8. At that point, IPTG was added to induce protein expression, with the induction carried out for 10 hours. After induction, the bacterial were harvested by centrifugation at 5000 rpm for 5 minutes and resuspended in lysis buffer containing protease inhibitors. The bacterial were disrupted via sonication to release the proteins, and the lysate was centrifuged to remove cell debris, retaining the supernatant for purification. Protein purification was achieved using a Ni-NTA affinity column, which binds the His-tagged proteins. The proteins were eluted stepwise with buffers containing different concentrations of imidazole. The purity of the eluted protein samples was verified using SDS-PAGE. If further concentration was needed, the protein was concentrated using ultrafiltration, followed by gel extraction, and finally dissolved in an appropriate buffer for storage.

***TTC and Masson Staining***

At 24 hours after MIR injury, the extent of myocardial tissue damage was assessed using 2,3,5-triphenyltetrazolium chloride (TTC) (T8170, Solarbio, Beijing) staining. Briefly, the heart was excised and sliced into myocardial sections (approximately 1 mm thick for mice and 5 mm thick for pigs). The sections were incubated in a 1% TTC solution at 37°C for 30 minutes. In the staining results, viable myocardium appeared red, while infarcted areas remained unstained and showed a greyish-white appearance. After staining, the area of each region was quantified using an image analysis system, and the infarct size was calculated as a percentage of the total myocardial area, providing a visual evaluation of the extent of MIR injury.

At the fourth week following the establishment of the MIR model, myocardial fibrosis was assessed using Masson's trichrome staining (G1346, Solarbio, Beijing). During the procedure, rat hearts were rinsed thoroughly with saline and fixed in 10% neutral-buffered formalin for 24 hours. Following fixation, the tissues were embedded in paraffin and sectioned into 5 μm-thick slices. The sections underwent standard dehydration and clearing procedures prior to staining. After staining, the distribution and density of collagen fibers (stained blue) were observed under a microscope to evaluate the extent of myocardial fibrosis.

***Ultrasound Assessment of Cardiac Physiological Function***

To evaluate cardiac physiological function in experimental animals, echocardiography was performed. During the procedure, both mice and Bama miniature pigs were anesthetized with isoflurane and subjected to mechanical ventilation. Cardiac function in mice and miniature pigs was assessed using the FUJIFILM Vevo 3100LT and Mindray Z70vet ultrasound systems, respectively. Pulsed-wave Doppler and M-mode echocardiography were employed to measure mitral valve flow parameters, enabling comprehensive evaluation of key cardiac physiological indicators, including left ventricular systolic and diastolic function.

***Electrophysiological Integration Assessment***

An optical mapping system was employed to evaluate cardiac electrical activity and excitation–contraction coupling in experimental animals. Prior to the procedure, animals were anticoagulated with an intraperitoneal injection of heparin sodium (3000 U/kg). After 15 minutes, anesthesia was induced using isoflurane. Following disinfection, the chest was opened by cutting along the midline of the ribcage after lifting the xiphoid process, exposing the heart. The heart was quickly excised and immersed in pre-cooled (4°C), calcium-free, modified Krebs buffer solution that had been equilibrated with 95% O₂ and 5% CO₂. The buffer contained NaCl, KCl, KH₂PO₄, MgSO₄, NaHCO₃, D-glucose, sodium pyruvate, and CaCl₂, with the pH adjusted to 7.35–7.45. The aorta was cannulated and secured with suture to a Langendorff perfusion system, ensuring no air bubbles entered the system. The heart was fixed in a silicone perfusion chamber and perfused retrogradely with oxygenated Krebs solution. After 10 minutes of equilibration and stabilization of cardiac rhythm, dye loading was initiated. Under dark conditions, Krebs solution was continuously perfused while Blebbistatin was introduced via a Y-connector to suppress mechanical contraction. After 1–2 minutes, cardiac motion ceased. Oxygen supply was then reduced, and part of the perfusate was removed. Pluronic F127 was added to the remaining solution and perfused for 10 minutes. Subsequently, the calcium indicator Rhod-2AM was slowly administered via the Y-connector and perfusion continued for another 15 minutes. Voltage-sensitive dye RH237 was then added. Finally, the reserved Krebs buffer was reintroduced to maintain the perfusion state. The heart was transferred to the imaging chamber. Red and blue electrodes were placed on the left ventricular apex and right atrium, respectively, and a black electrode was grounded to complete ECG signal acquisition setup. Data acquisition began after adjusting sampling parameters for continuous monitoring. The optical system was focused on the cardiac surface, and the lens was adjusted to obtain optimal image field and resolution. Action potentials and calcium transients under sinus rhythm were recorded three times. A pacing electrode was inserted ~1 mm into the right ventricle. Initial parameters were set to 6 Hz frequency, 2 ms pulse width, and the stimulation current was gradually increased from 0 mA until ECG signals synchronized with the stimulus, determining the diastolic threshold current. Using twice the threshold current, action potential and calcium signals were recorded at 6 Hz pacing. A programmed electrical stimulation (S1–S2) protocol was performed, consisting of eight consecutive S1 stimuli followed by a premature S2 stimulus. The S1–S1 interval was set 20 ms shorter than the baseline RR interval, and the S1–S2 interval was reduced in 5 ms increments until the QRS complex could no longer be elicited. The final S1–S2 interval was recorded as the ventricular effective refractory period (ERP). To induce arrhythmias, high-frequency (50 Hz) burst pacing was applied at stepwise current levels of 5, 10, 15, and 20 mA, with 3-minute intervals. The incidence, duration of ventricular arrhythmias, and the ventricular fibrillation threshold were recorded. Throughout the experiment, ECGs under sinus rhythm, pacing, and arrhythmia induction were collected, alongside changes in membrane potential and calcium transients. Comparisons of pre- and post-I/R cardiac electrical activity, including depolarization/repolarization sequences, action potential duration, and calcium transient dynamics, enabled comprehensive assessment of excitation–contraction coupling and cardiac rhythm stability.

***Molecular Docking Simulation***

Molecular docking simulations were performed using AutoDock to investigate the binding interactions between the CBX7 protein and small-molecule compounds. The three-dimensional structure of CBX7 was first retrieved, and the structures of the small-molecule ligands were prepared accordingly. A docking grid was then defined around the predicted binding site, and docking simulations were carried out. The binding energies were analyzed using PyMOL, and protein–ligand interactions were visualized. Key hydrogen bonds and hydrophobic interactions were identified, and the distances between interacting residues and ligand moieties were measured. This analysis provided insights into the binding mechanism between CBX7 and the small molecules, offering a theoretical foundation for subsequent drug design efforts.

***High-Throughput Compound Screening***

The three-dimensional structure of the Cbx7 protein (UniProt ID: B0QYP2) was downloaded from the UniProt database (<https://www.uniprot.org>) and subjected to energy minimization to optimize its conformation and improve the accuracy of molecular modeling. A compound library comprising approximately 4,566 compounds was obtained from Topscience, and the database was established based on specific chemical properties and known bioactivities. Each compound was docked into the predicted binding site of CBX7 using molecular docking techniques. Key parameters, including binding energy, number of hydrogen bonds, and hydrophobic interactions, were analyzed to identify compounds with high binding affinity. To enhance virtual screening accuracy, the PLANET model—a graph neural network-based prediction tool—was introduced to evaluate the relationship between molecular structure and biological activity. Based on a comprehensive evaluation combining docking scores and PLANET predictions, the top 50 candidate compounds were selected. SPR experiments were performed to assess the binding kinetics of these compounds with CBX7, and compounds with dissociation constant (KD) values below 5 μM were identified.

In the in vitro phase, primary cardiomyocytes and cardiac fibroblasts (CMFs) were isolated from mouse hearts. Cardiomyocytes were cultured in DMEM/F12 medium, while CMFs were maintained in DMEM medium; both media were supplemented with 10% fetal bovine serum and antibiotics, and cultured at 37°C in a 5% CO₂ incubator. After simulating the MIR environment in vitro, each candidate compound was added to the culture medium at a final concentration of 5 μM and incubated for 24 hours.

Cell viability was assessed using the CCK8 assay following the manufacturer’s instructions, with blank and control groups included for reliability. The results revealed that two compounds significantly enhanced cardiomyocyte viability, up to 1.5-fold compared to controls, suggesting a strong potential in promoting cardiomyocyte survival and functional recovery. This may involve the regulation of cellular metabolism, antioxidant responses, or apoptosis-related signaling pathways. Notably, the viability of CMFs increased by no more than 1.1-fold, indicating favorable cell specificity and targeting ability.

Subsequently, a mitochondrial stress test (Mito Stress Test) was performed on these two candidate compounds to further evaluate their effects on mitochondrial function. Measurement of oxygen consumption rate (OCR) demonstrated that one compound significantly improved basal respiration, ATP production, and maximal respiration capacity, suggesting enhanced metabolic activity. This compound, identified as δ-Amyrenone (δAe), was ultimately selected as a functional CBX7 inhibitor due to its strong activity, selectivity, and mitochondrial protective effects for subsequent in vivo investigations.

***Fluorescence Recovery After Photobleaching (FRAP)***

Prior to imaging, cells were subjected to various experimental treatments. FRAP experiments were performed using a ZEISS LSM 980 confocal microscope. A region of interest (ROI) exhibiting GFP or mCherry fluorescence was selected and completely photobleached using a high-intensity laser. Following photobleaching, fluorescence recovery within the ROI was monitored in real time, and data were collected at defined time intervals to evaluate the dynamic movement and redistribution of fluorescently labeled proteins. Fluorescence intensity was recorded both before bleaching and during the early phase of recovery to systematically analyze the diffusion behavior and kinetic characteristics of fluorescent proteins under different treatment conditions. This method provides insights into the intracellular mobility of proteins and their interactions with changes in the local microenvironment.

***Droplet Assay In Vitro***

To evaluate liquid–liquid phase separation (LLPS) in vitro, purified CBX7-GFP and ATP7A-mCherry proteins were diluted to designated concentrations and mixed in a phase separation buffer containing 20 mM Tris-HCl (pH 7.5), 75 mM KCl, 10% BSA, 5% PEG, and 1 mM DTT. The protein mixture was incubated at 37°C for 10 minutes to promote droplet formation, with gentle mixing to ensure uniform distribution of proteins throughout the solution. Following incubation, samples were imaged using a fluorescence microscope to assess the formation of droplet-like structures. This assay allows for the visualization and analysis of protein–protein interactions and their capacity for phase separation under defined in vitro conditions, providing a straightforward model to study LLPS behavior.

***Serum Biochemistry and Cardiac Histological Analysis***

Peripheral blood samples were collected from experimental animals and submitted to Lilai biomedicine for serum biochemical analysis. The assays included liver and kidney function parameters such as γ-glutamyl transferase (γ-GT), aspartate aminotransferase (AST), uric acid (UA), and blood urea nitrogen (UREA). At 4 weeks post-MIR, all mice and pigs were euthanized. Major organs were harvested, and paraffin sections of 5 μm thickness were prepared for hematoxylin and eosin (HE) staining to assess potential systemic toxicity. Additionally, heart tissues were sectioned at 1 mm thickness for mice and 5 mm for pigs to evaluate and document the infarct size macroscopically.

***Seahorse Metabolic Function Analysis***

To evaluate mitochondrial function and glycolytic capacity of isolated cardiomyocytes, the Seahorse XF Analyzer (Agilent, USA) was used to measure the oxygen consumption rate (OCR) and extracellular acidification rate (ECAR). Prior to the experiment, Seahorse XFe96 microplates (102959-100, Agilent, USA) were coated with Cell-Tak at a concentration of 22 μg/ml (pH 6.5–8) and incubated at 37°C for 20 minutes to promote cell adhesion. After neutralization, the plates were washed twice with deionized water, air-dried, and stored at 4°C until use. Isolated cardiomyocytes were seeded onto the pre-treated plates and allowed to adhere overnight in a 37°C incubator. On the following day, the culture medium was replaced with pre-warmed, low-buffered DMEM/F12, and the cells were incubated for 1 hour in a CO₂-free incubator. The sensor cartridge was hydrated with Seahorse XF Calibrant one day prior to the assay. For OCR measurement, the Seahorse XF Cell Mito Stress Test Kit (103015-100, Agilent, USA) was used following the manufacturer’s protocol. The compounds were sequentially injected into the wells as follows: oligomycin (4 μM), FCCP (4 μM), and a mixture of rotenone (2 μM) and antimycin A (2 μM) to induce mitochondrial stress response. For ECAR detection, the XF Glycolysis Stress Test Kit (103020-100, Agilent, USA) was used. Glucose (20 mM), oligomycin (4 μM), and 2-deoxyglucose (2-DG, 100 mM) were sequentially injected to stimulate glycolytic activity. Each reagent was loaded in a volume of 25 μl into the designated injection ports of the sensor cartridge. The assay was run using alternating cycles of 3 minutes mixing and 3 minutes measuring. Data were acquired using the XF96 Analyzer and normalized to total protein content per well to ensure accuracy and comparability across samples.

***TUNEL Staining***

TUNEL staining was performed to evaluate apoptotic cell death in myocardial tissue. Paraffin-embedded heart sections were first deparaffinized and rehydrated using a standard protocol, including incubation in xylene, followed by a graded ethanol series and final hydration in distilled water. To increase tissue permeability, 20 μg/mL DNase-free proteinase K was applied to the sections and incubated at room temperature for 30 minutes. After incubation, the sections were washed three times with PBS. The TUNEL reaction mixture was prepared according to the instructions provided with the One-Step TUNEL Apoptosis Assay Kit (C1086, Beyotime, China). Sections were incubated with the TUNEL working solution at 37°C for 60 minutes in the dark, allowing terminal deoxynucleotidyl transferase (TdT) to incorporate fluorescently labeled dUTP at the 3’-OH ends of fragmented DNA. After staining, sections were washed three times with PBS and counterstained with DAPI to visualize nuclei. DAPI staining was performed at room temperature for 5 minutes in the dark, followed by PBS rinsing and coverslip mounting. Images of TUNEL-positive cells and DAPI-stained nuclei were captured using a fluorescence microscope. The images were saved for subsequent quantitative analysis to assess the level of apoptosis in myocardial tissue.

***Establishment and Validation of an Aged Miniature Pig Model***

To establish an aged miniature pig model, healthy 8-week-old Bama miniature pigs (average body weight: 15–20 kg) were selected and intraperitoneally injected with D-galactose at a dosage of 200 mg/kg/day for 8 consecutive weeks. This intervention was intended to induce aging-like phenotypes, including oxidative stress, chronic inflammation, and impaired cardiac function, thereby mimicking natural aging. During the modeling period, body weight, food intake, and general health status were closely monitored to ensure the well-being of the animals and the success of the aging induction. Following the modeling period, the aging phenotype was validated through serum biochemical assays and histological analysis of cardiac tissue. Specific assessments included: Oxidative stress evaluation: Peripheral blood samples were collected to measure serum levels of malondialdehyde (MDA) and superoxide dismutase (SOD), reflecting oxidative damage and antioxidant capacity, respectively. Inflammatory and nutritional status: Serum interleukin-6 (IL-6) and insulin-like growth factor-1 (IGF-1) levels were measured to assess systemic inflammation and age-related metabolic changes. Cardiac structural and functional assessment: Echocardiographic measurement of left ventricular anterior wall thickness during diastole (LVAWd) was performed to evaluate age-associated alterations in cardiac morphology and function. Histological confirmation: Co-immunofluorescence staining for cardiac troponin I (cTnI) and wheat germ agglutinin (WGA) was conducted to assess cardiomyocyte morphology and tissue organization, further confirming myocardial aging features.

**Supplementary figures**


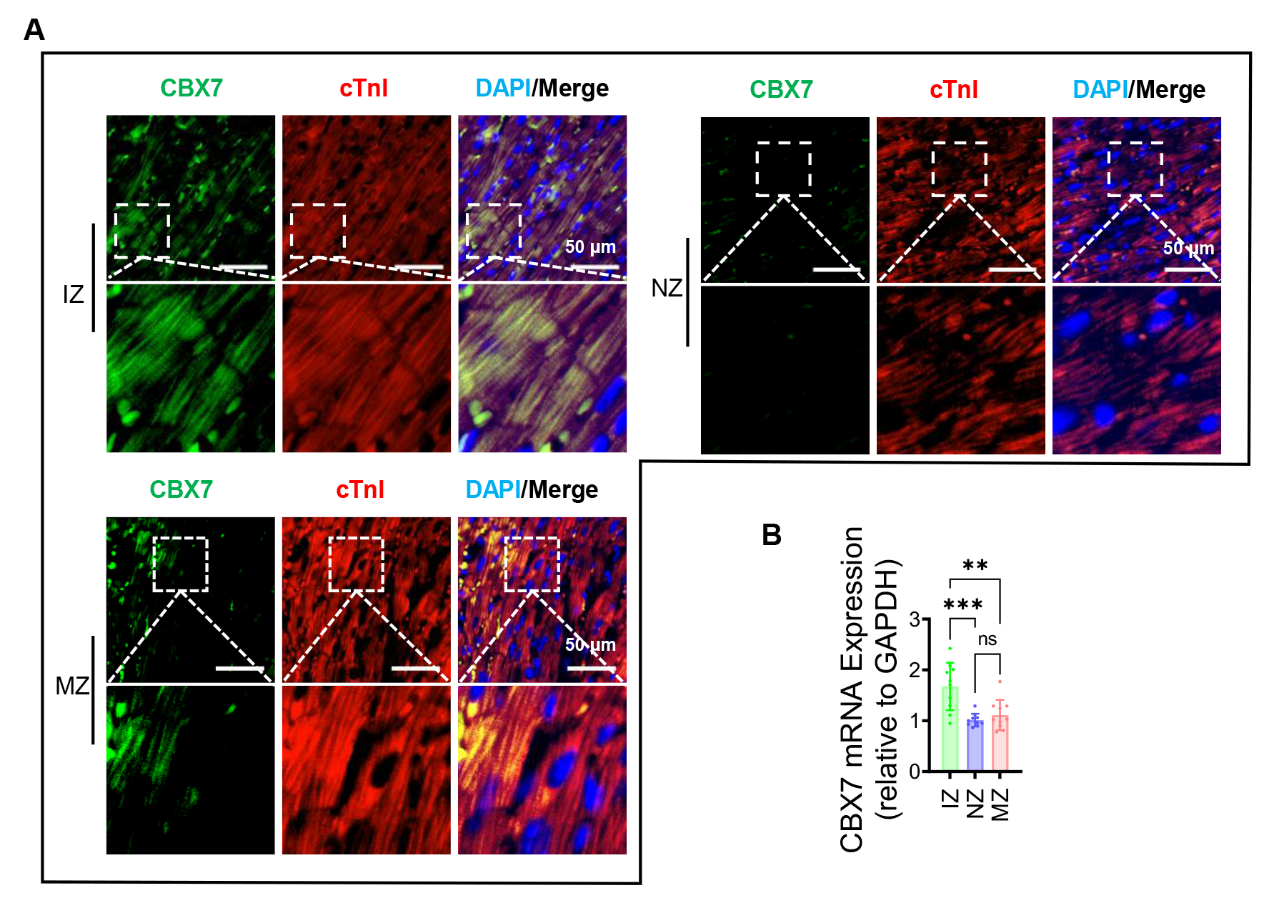


**Figure S1. Regional increase of CBX7 in aged hearts at 1 day post-MIR.** Aged mice were subjected to MIR. Hearts were harvested at 24 h and sectioned to sample the infarct zone (IZ), marginal zone (MZ), and normal zone (NZ). **(A)** Representative images show CBX7 (green) within cardiomyocytes identified by cTnI (red) and nuclei by DAPI (blue) (scale bar, 50 μm). CBX7 signal is highest in IZ, intermediate in MZ, and lowest in NZ, with perinuclear and cytoplasmic enrichment in IZ cardiomyocytes. Regions of interest were sampled across multiple fields per section and averaged per animal to minimize field bias. **(B)** CBX7 mRNA measured from microdissected IZ, MZ, and NZ and normalized to GAPDH shows the same gradient (IZ > MZ > NZ), indicating coordinated transcriptional and protein-level induction at the injury core and border. Data are mean ± SD (n = 10 biological replicates). One-way ANOVA with Tukey post hoc test, **p < 0.01 where indicated; ns, not significant. **Abbreviations.** MIR, myocardial ischemia–reperfusion; IZ, infarct zone; MZ, marginal zone; NZ, normal zone; cTnI, cardiac troponin I.


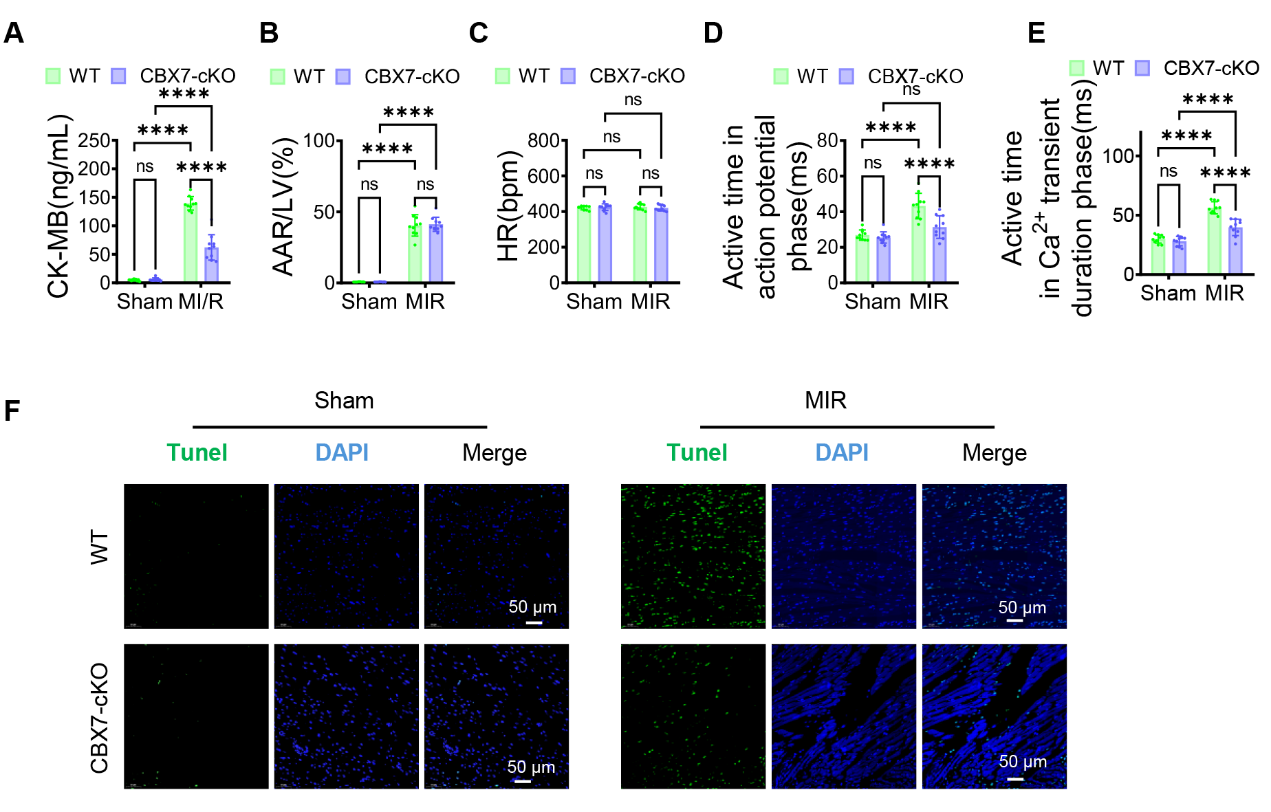


**Figure S2. Auxillary injury indices, rhythm, and cell death in aged MIR.** Aged mice underwent myocardial ischemia–reperfusion (MIR) with or without cardiomyocyte-specific CBX7 deletion (CBX7-cKO). Measurements were obtained at 24 h (biomarkers, TUNEL) and in the subacute window (optical mapping). **(A)** Serum CK-MB at 24 h is lower in CBX7-cKO versus control, indicating reduced myonecrosis. **(B)** Area at risk (AAR) as % LV (from TTC-defined risk regions) shows no significant difference between groups, confirming that infarct-sparing in CBX7-cKO is not due to smaller ischemic territory. **(C)** Heart rate is comparable between groups, indicating that subsequent electrical differences are not driven by baseline chronotropy. **(D)** Activation-time maps from optical action potentials illustrate more uniform conduction with fewer slow-conduction or block regions in CBX7-cKO relative to controls. **(E)** Ca²⁺-transient duration maps show shorter and less heterogeneous calcium handling in CBX7-cKO, consistent with improved excitation–contraction coupling and lower arrhythmogenic substrate. **(F)** TUNEL staining (green) with DAPI (blue) at 24 h demonstrates fewer apoptotic nuclei in CBX7-cKO myocardium (scale bar, 50 μm), aligning with reduced biomarker release.Data are mean ± SD (n = 10 per group unless indicated). one-way ANOVA with Tukey post hoc test. ****p < 0.001; ns, not significant. **Abbreviations.** MIR, myocardial ischemia–reperfusion; LV, left ventricle; CK-MB, creatine kinase–MB; AAR, area at risk; TTC, triphenyltetrazolium chloride.


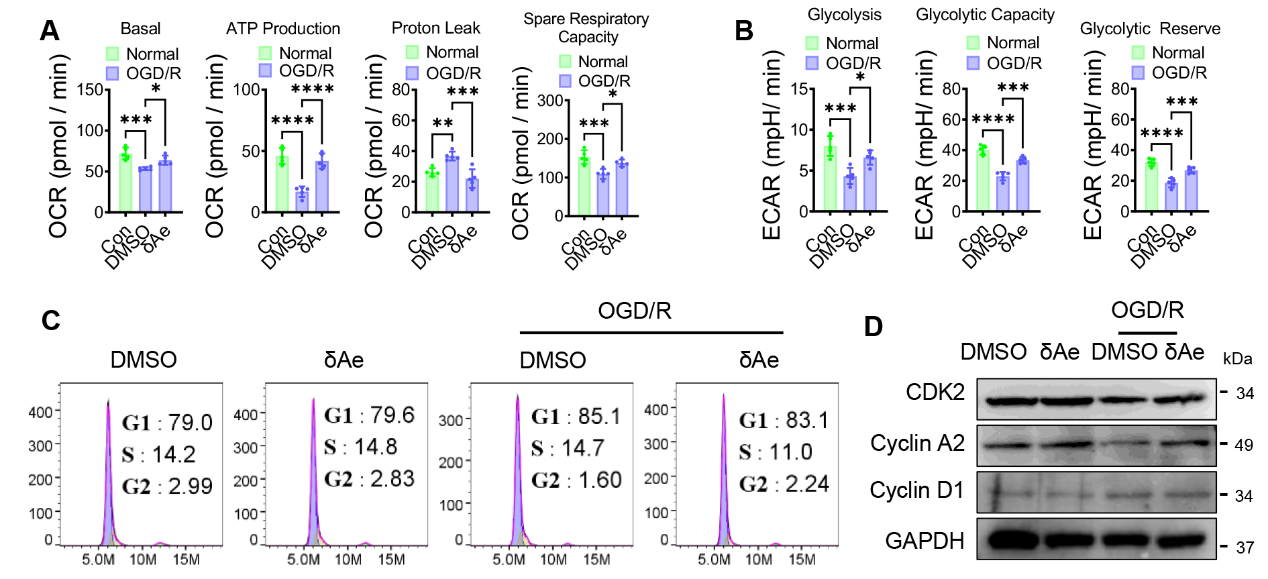


**Figure S3. Bioenergetic profiling and cell-cycle readouts in aged cardiomyocytes.** Primary aged mouse cardiomyocytes were subjected to oxygen–glucose deprivation followed by reoxygenation (OGD/R) and treated with vehicle or δAe (non-toxic, protective dose selected from viability screens). Seahorse assays and cell-cycle readouts were obtained from independent experiments. **(A)** OCR-derived parameters calculated from sequential additions of oligomycin, FCCP, and rotenone plus antimycin A. OGD/R depresses basal respiration, ATP-linked respiration, and spare respiratory capacity, while increasing proton leak. δAe partially restores maximal and spare capacity, improves ATP production, and normalizes proton leak, indicating preserved mitochondrial reserve and tighter coupling. **(B)** ECAR-derived parameters calculated from sequential additions of glucose, oligomycin, and 2-deoxy-D-glucose. OGD/R blunts glycolysis, glycolytic capacity, and glycolytic reserve; δAe improves all three, mitigating the glycolytic bottleneck that follows ischemic stress. **(C)** Representative cell-cycle histograms (G1/S/G2) show that aged cardiomyocytes remain predominantly G1-arrested after OGD/R; δAe does not induce a shift toward S- or G2-phase, consistent with a non-proliferative protection mode in aged cells. **(D)** Western blots of Cyclin D1, Cyclin A2, and CDK2 show no overt upregulation with δAe under OGD/R, further supporting bioenergetic rescue without cell-cycle re-entry. Data are mean ± SD from independent experiments (sample sizes indicated in panels). One-way ANOVA with Tukey’s post hoc test. *p < 0.05, **p < 0.01, ***p < 0.005, ****p < 0.001. **Abbreviations.** δAe, δ-Amyrenone; OGD/R, oxygen–glucose deprivation and reoxygenation; OCR, oxygen consumption rate; ECAR, extracellular acidification rate; FCCP, carbonyl cyanide-p-trifluoromethoxyphenylhydrazone; 2-DG, 2-deoxy-D-glucose; ATP, adenosine triphosphate; CDK2, cyclin-dependent kinase 2.


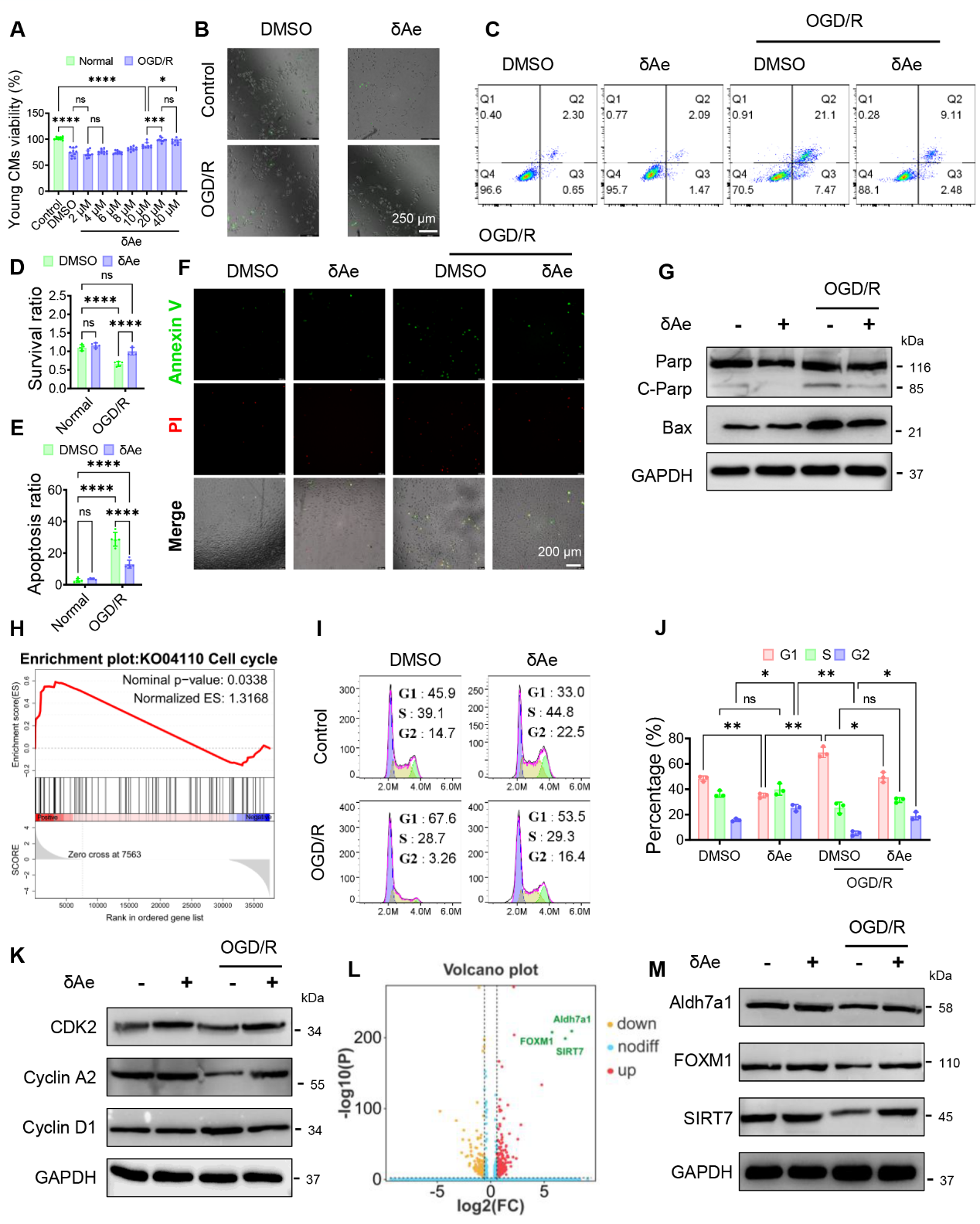


**Figure S4. δAe promotes survival and cell-cycle progression in young cardiomyocytes under OGD/R. (A)** CCK-8 viability across δAe concentrations identifies a non-toxic, pro-survival window (peak efficacy at 20 μM), which is used in subsequent assays. **(B)** Representative YP-1 staining after treatments (scale bar, 250 μm) shows improved cell integrity with δAe. **(C-E)** Annexin V/PI flow cytometry and quantification demonstrate increased survival and reduced apoptosis with δAe following OGD/R. **(F)** Annexin V/PI fluorescence images (scale bar, 200 μm) corroborate flow-cytometric findings. **(G)** Western blots show decreased cleaved PARP (C-PARP) and Bax with δAe, consistent with apoptosis suppression. **(H)** GSEA reveals significant enrichment of the Cell Cycle pathway after δAe under OGD/R, indicating transcriptional activation of mitotic programs. **(I, J)** Cell-cycle histograms and pooled phase analysis show relief of G1 arrest with δAe and a corresponding increase in S/G2 populations. **(K)** Western blots confirm upregulation of Cyclin D1, CDK2, and Cyclin A2, supporting accelerated G1–S transition and S-phase progression. **(L)** Volcano plot of differentially expressed genes highlights δAe-responsive transcripts.
**(M)** Western-blot validation confirms induction of Aldh7a1, FOXM1, and SIRT7, consistent with enhanced cell-cycle competence and stress resilience. Data are mean ± SD from independent experiments (sample sizes indicated in panels). One-way ANOVA with Tukey’s post hoc test. *p < 0.05, **p < 0.01, ***p < 0.005, ****p < 0.001; ns, not significant. **Abbreviations.** δAe, δ-Amyrenone; OGD/R, oxygen–glucose deprivation and reoxygenation; CCK-8, Cell Counting Kit-8; YP-1, cell-permeant viability dye; Annexin V/PI, Annexin V–propidium iodide; PARP, poly(ADP-ribose) polymerase; C-PARP, cleaved PARP; Bax, BCL-2–associated X protein; GSEA, gene set enrichment analysis; CDK2, cyclin-dependent kinase 2.


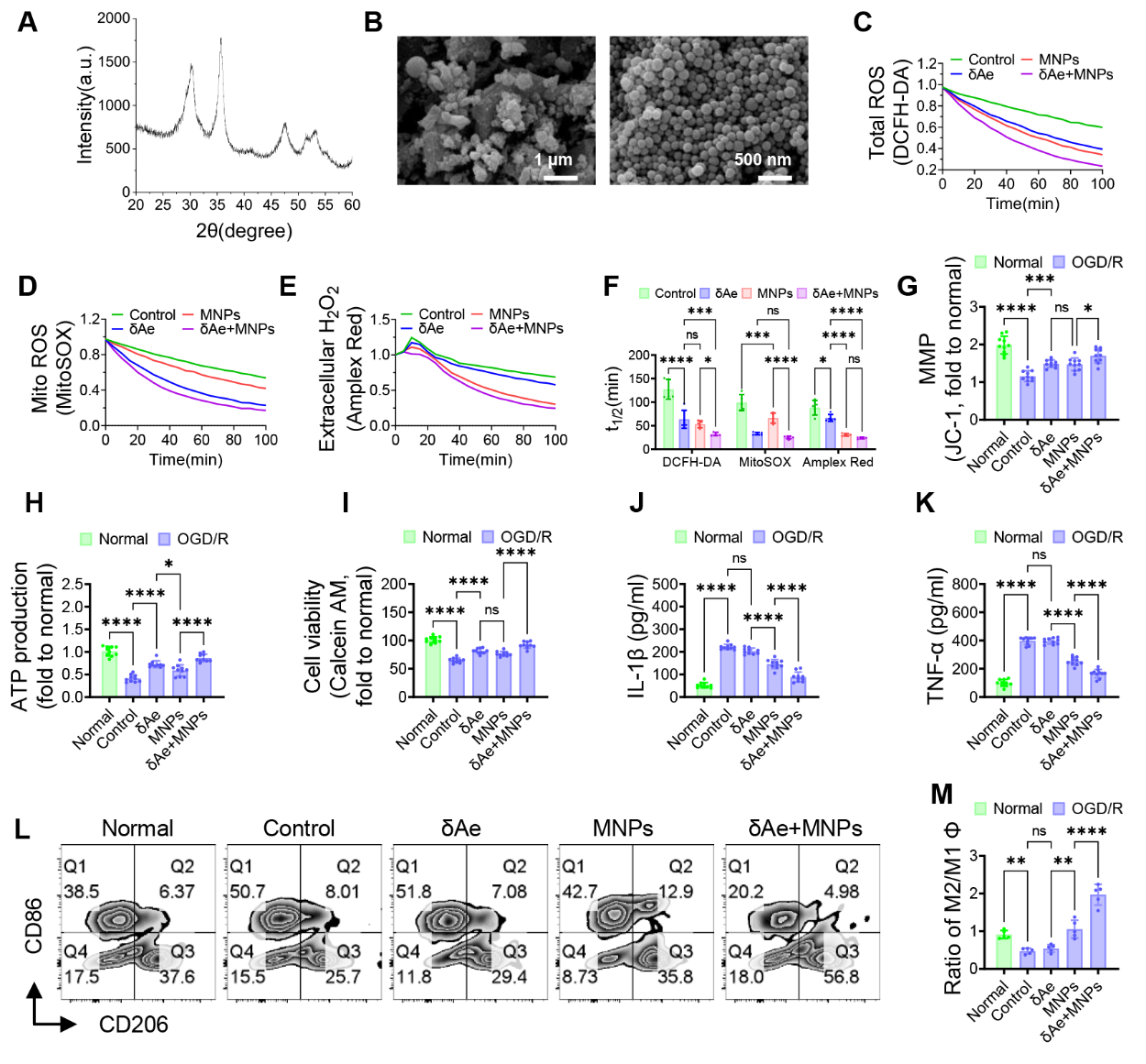


**Figure S5. Roles and synergy of δAe and MNPs under OGD/R in aged cardiomyocytes.** **(A)** X-ray diffraction of MNPs shows the broad amorphous signature characteristic of melanin-like nanoparticles. **(B)** SEM reveals spherical, uniformly dispersed MNPs (scale bars, 1 μm and 500 nm). **(C)** Total intracellular ROS kinetics (DCFH-DA) in aged cardiomyocytes under OGD/R show that δAe plus MNPs achieves the fastest and most complete suppression. **(D)** Mitochondrial superoxide kinetics (MitoSOX) show an analogous advantage for the combination over either monotherapy. **(E)** Extracellular H₂O₂ kinetics (Amplex Red) likewise demonstrate the greatest and most rapid reduction with δAe plus MNPs. **(F)** Summary half-lives (t₁/₂) for panels C-E confirm significantly shorter decay times with the combination than with δAe or MNPs alone. **(G)** JC-1 assays indicate better preservation of mitochondrial membrane potential with δAe plus MNPs. **(H)** ATP production is elevated by the combination relative to single agents. **(I)** Calcein-AM viability is highest with δAe plus MNPs, consistent with improved redox control and mitochondrial function. **(J)** ELISA for IL-1β shows greater suppression with the combination than with either agent alone. **(K)** ELISA for TNF-α shows a similar reduction pattern. **(L)** Flow cytometry of CD86 and CD206 in aged CM–macrophage co-culture at a 4:1 ratio demonstrates a shift toward CD206⁺ cells with δAe plus MNPs. **(M)** The M2/M1 ratio increases significantly under the combination treatment, indicating enhanced pro-repair polarization. Data are mean ± SD. One-way ANOVA with Tukey’s post hoc test. *p < 0.05, **p < 0.01, ***p < 0.005, ****p < 0.001; ns, not significant. **Abbreviations.** OGD/R, oxygen–glucose deprivation and reoxygenation; CM, cardiomyocyte; MNPs, melanin nanoparticles; DCFH-DA, 2′,7′-dichlorodihydrofluorescein diacetate; MitoSOX, mitochondrial superoxide indicator; Amplex Red, resorufin-based H₂O₂ readout; t₁/₂, half-life; JC-1, 5,5′,6,6′-tetrachloro-1,1′,3,3′-tetraethylbenzimidazolylcarbocyanine iodide; ELISA, enzyme-linked immunosorbent assay; IL-1β, interleukin-1 beta; TNF-α, tumor necrosis factor alpha.


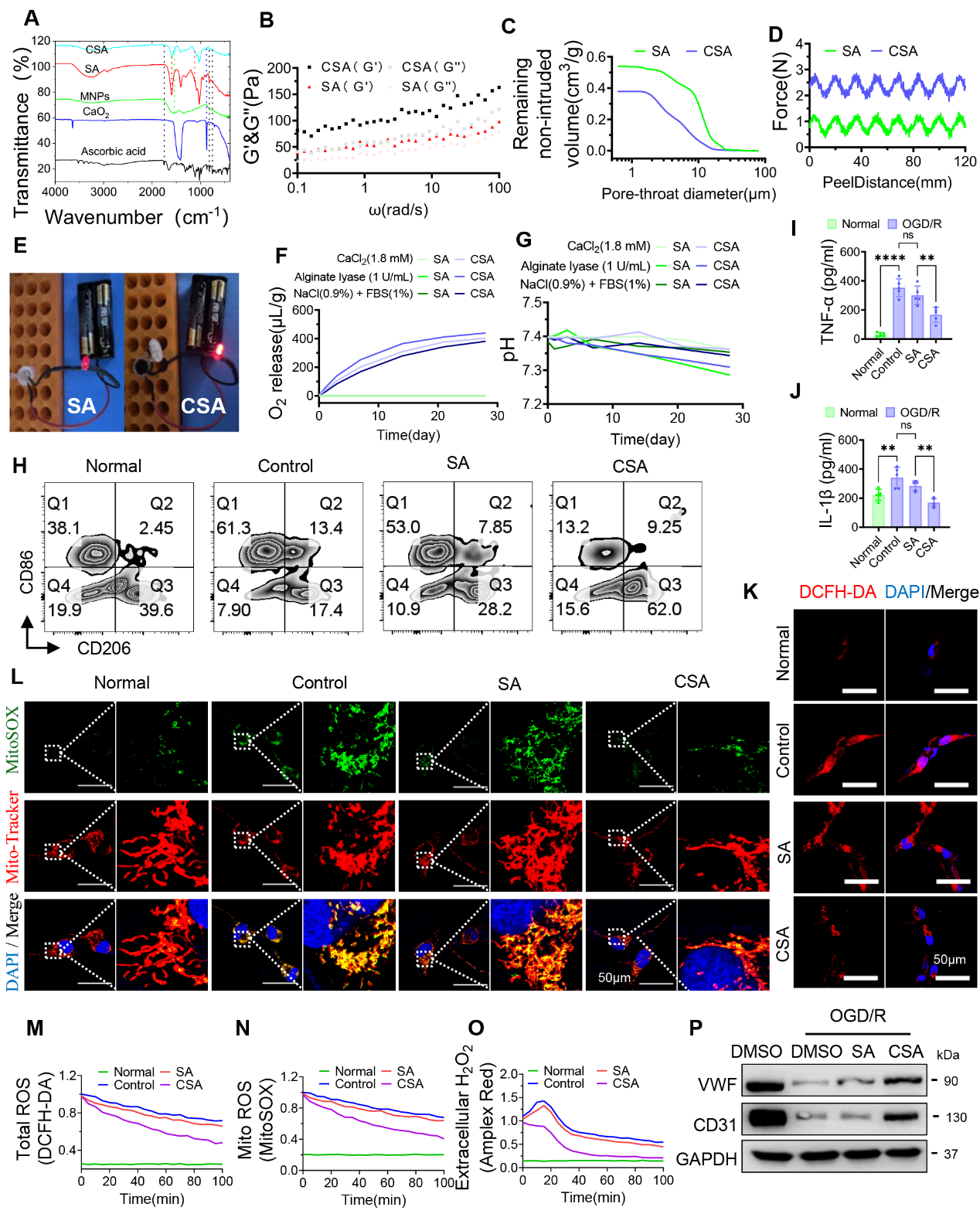


**Figure S6. SA vs. CSA: materials, mechanics, and biofunction. (A)** FTIR spectra of individual components (alginate, CaO₂ nanoparticles, melanin nanoparticles, ascorbic acid) and composite gels (SA, CSA) confirm successful incorporation without adverse new peaks. **(B)** Pre-injection rheology shows pronounced shear-thinning for both SA and CSA, with CSA maintaining higher low-frequency viscosity yet flowing readily under shear-supporting catheter delivery prior to in situ gelation. **(C)** Mercury intrusion porosimetry (non-intruded volume vs pore diameter) indicates a shift toward smaller pore throats and a narrower distribution in CSA, consistent with a denser network. **(D)** Representative 180° peel force–distance traces show more stable interfacial debonding for CSA. **(E)** LED demonstration indicates higher conductivity for CSA than SA. **(F)** Dissolved O₂ release profiles reveal sustained oxygenation from CSA over an extended window. **(G)** pH during degradation remains near physiological range for CSA, indicating effective buffering. **(H)** Macrophage polarization assessed in co-culture of aged cardiomyocytes and macrophages at a 4:1 CM:Mac ratio under OGD/R shows a shift toward CD206⁺ cells with CSA (flow cytometry for CD86/CD206), indicating pro-repair polarization. **(I, J)** In aged cardiomyocyte monocultures under OGD/R, ELISAs show CSA reduces TNF-α and IL-1β compared with SA and control. **(K)** DCFH-DA fluorescence images (scale bars, 50 μm) show lower total intracellular ROS with CSA. **(L)** MitoSOX/MitoTracker/DAPI images (scale bars, 50 μm) indicate diminished mitochondrial superoxide with CSA. **(M–O)** Real-time ROS kinetics in aged cardiomyocytes demonstrate that CSA accelerates and deepens redox control versus SA across (M) total intracellular ROS (DCFH-DA), (N) mitochondrial ROS (MitoSOX), and (O) extracellular H₂O₂ (Amplex Red). **(P)** In endothelial cells, Western blots show higher VWF and CD31 with CSA than SA, supporting pro-angiogenic potential. Data are mean ± SD; one-way ANOVA with Tukey’s post hoc test. **p < 0.01, **p < 0.001; ns, not significant. **Abbreviations.** SA, alginate hydrogel; CSA, composite alginate hydrogel (alginate + CaO₂ nanoparticles + melanin nanoparticles + ascorbic acid); FTIR, Fourier-transform infrared spectroscopy; OGD/R, oxygen–glucose deprivation and reoxygenation; CM, cardiomyocyte; Mac, macrophage; LED, light-emitting diode; DCFH-DA, 2′,7′-dichlorodihydrofluorescein diacetate; MitoSOX, mitochondrial superoxide indicator; VWF, von Willebrand factor; CD31, platelet endothelial cell adhesion molecule-1.


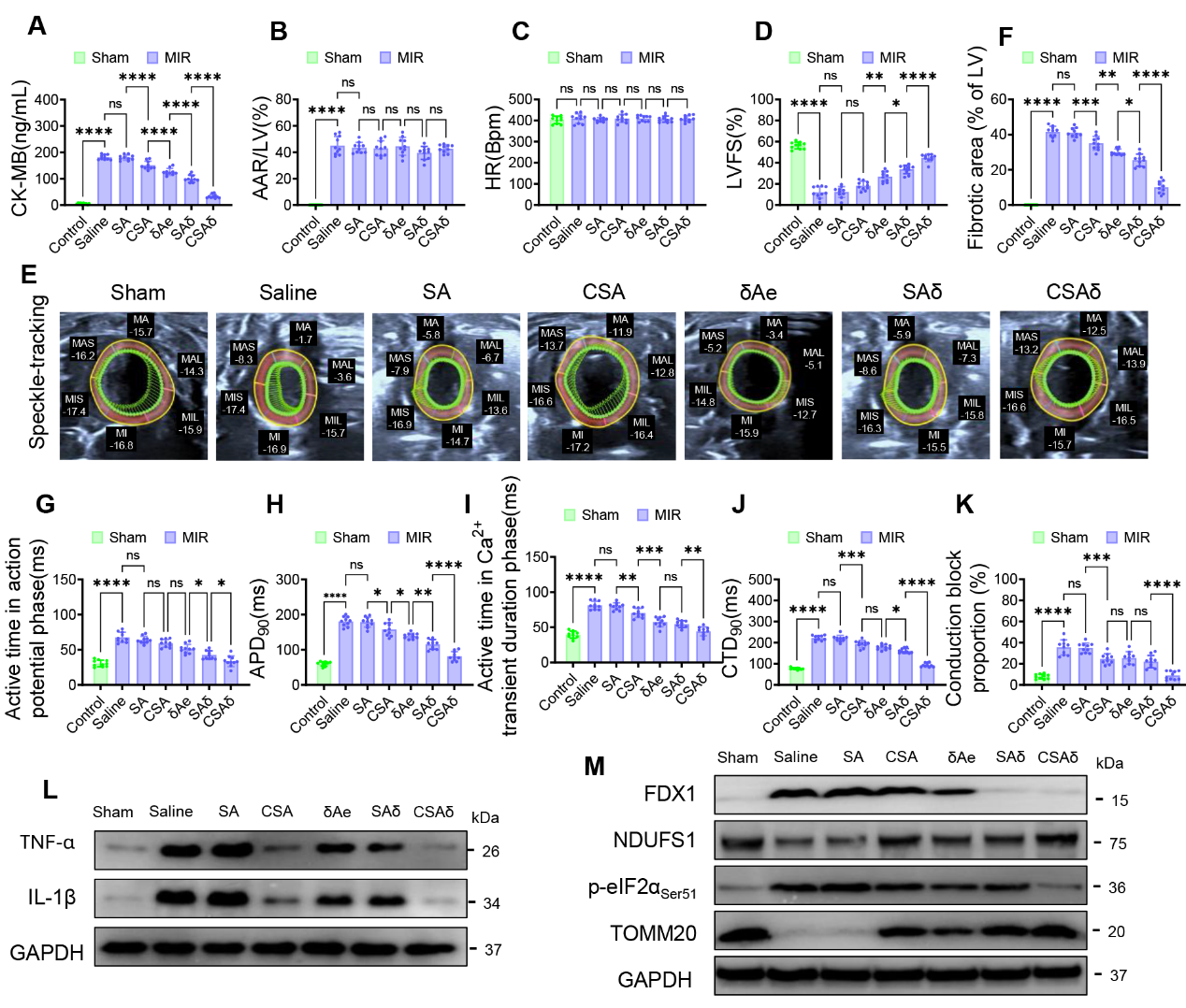


**Figure S7. Functional, fibrotic, electrical, and molecular readouts across treatments in aged mice MIR.** Aged C57BL/6J mice were randomized to Sham, Saline, SA, CSA, δAe, SAδ, or CSAδ (n = 10/group). Acute assays were performed at 24 h (injury biomarkers and cytokines), cuproptosis and mitochondrial markers at 48 h, and echocardiography, histology, and optical electrophysiology at 4 weeks. **(A)** CK-MB shows the lowest circulating injury with CSAδ. **(B)** AAR/LV (%) is comparable among MIR groups, indicating infarct sparing is not due to smaller ischemic territories. **(C)** Heart rate (bpm) is similar across groups. **(D)** LVFS (%) improves with CSAδ, exceeding SAδ and δAe. **(E)** Speckle-tracking bull’s-eye (segmental circumferential strain) demonstrates recovery of ischemia-affected segments with CSAδ. **(F)** Fibrotic area (% LV; Masson’s) is reduced by CSAδ. **(G)** Activation-window duration (ms) shifts toward physiologic ranges with CSAδ. **(H)** APD90 (ms) shortens with CSAδ, reflecting normalized repolarization. **(I)** Ca²⁺-transient active time (ms) decreases with CSAδ, indicating improved Ca²⁺ handling. **(J)** CTD90 (ms) shortens with CSAδ, supporting restored Ca²⁺ reuptake. **(K)** Conduction-block proportion (%) is lower in CSAδ, suggesting a less arrhythmogenic substrate. **(L)** Cardiac TNF-α and IL-1β (WB) are reduced in CSAδ, consistent with early anti-inflammatory effects. **(M)** Cuproptosis and mitochondrial markers (FDX1, p-eIF2α_Ser51_, TOMM20, NDUFS1; WB) indicate decreased cuproptosis signaling and recovery of mitochondrial structure/function with CSAδ. Data are mean ± SD (n = 10). One-way ANOVA with Tukey’s post hoc test; *p < 0.05, **p < 0.01, ***p < 0.005, ****p < 0.001; ns, not significant. **Abbreviations.** MIR, myocardial ischemia–reperfusion; AAR/LV, area at risk over left ventricle; LVFS, left ventricular fractional shortening; APD90, action potential duration at 90% repolarization; CTD90, Ca²⁺-transient duration to 90% recovery; WB, western blot; FDX1, ferredoxin-1; p-eIF2α^Ser51, phospho-eukaryotic initiation factor 2α (Ser51); TOMM20, translocase of outer mitochondrial membrane 20; NDUFS1, NADH:ubiquinone oxidoreductase core subunit S1.


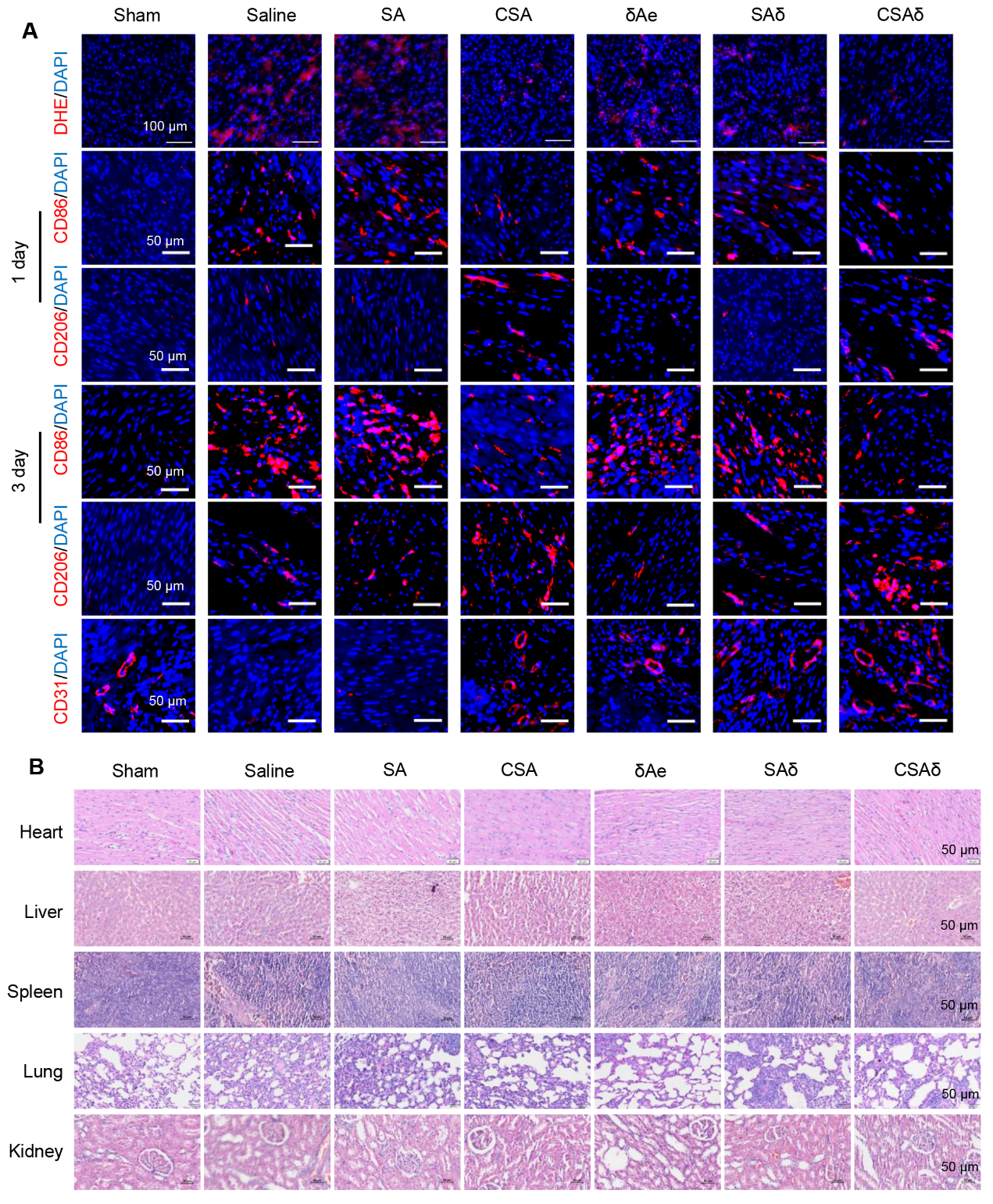


**Figure S8. Immunofluorescence and systemic safety. (A)** Representative immunofluorescence panels from peri-infarct myocardium at day 1 and day 3 show: reduced DHE signal (ROS) with treatment; decreased CD86 (M1) and increased CD206 (M2), indicating a shift toward a reparative phenotype; and higher CD31 staining consistent with increased microvessel density. Nuclei are counterstained with DAPI. Scale bars as indicated. **(B)** Hematoxylin and eosin (H&E) sections of heart, liver, spleen, lung, and kidney at the study endpoint reveal preserved tissue architecture without overt necrosis, inflammatory infiltrates, or structural injury, supporting in vivo biosafety. Data are representative of biological replicates; quantitative analyses are provided in the main and supplementary figures where applicable. **Abbreviations.** IF, immunofluorescence; DHE, dihydroethidium; DAPI, 4′,6-diamidino-2-phenylindole; CD31, platelet endothelial cell adhesion molecule-1; H&E, hematoxylin and eosin.


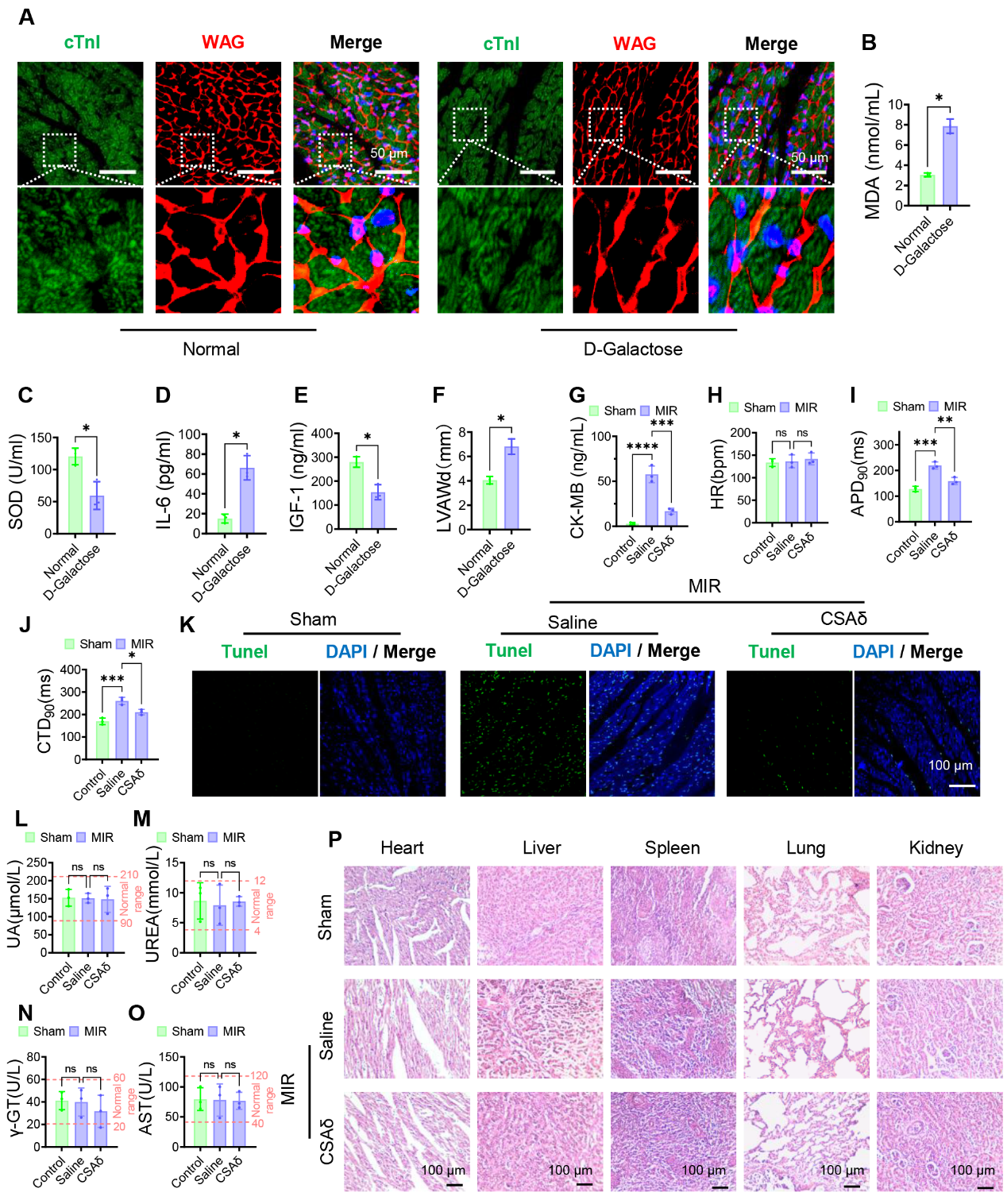


**Figure S9. Model validation, extended efficacy, and safety in aged pigs. (A)** WGA/cTnI/DAPI immunofluorescence from left ventricular myocardium shows enlarged cardiomyocyte cross-sectional areas in D-galactose–treated (aged) pigs compared with normal pigs (scale bars, 50 μm), validating the hypertrophic phenotype. **(B-E)** Systemic biomarkers confirm aging status: malondialdehyde (MDA, lipid peroxidation) and interleukin-6 (IL-6, inflammation) are elevated, whereas superoxide dismutase (SOD, antioxidant enzyme) and insulin-like growth factor-1 (IGF-1, anabolic signaling) are reduced. **(F)** Echocardiography indicates reduced left ventricular anterior wall thickness at end-diastole (LVAWd, mm) in aged pigs, consistent with structural degeneration. After MIR and treatment during reperfusion, **(G)** serum CK-MB at 24 h is lower with CSAδ versus saline, indicating reduced acute myonecrosis; **(H)** heart rate at follow-up is comparable between groups. At 4 weeks, optical mapping shows electrophysiologic improvement with CSAδ: **(I)** action potential duration at 90% repolarization (APD90) and **(J)** Ca²⁺ transient duration at 90% recovery (CTD90) are shortened toward physiologic ranges. **(K)** TUNEL immunofluorescence demonstrates fewer apoptotic nuclei in CSAδ-treated myocardium versus saline (scale bar, 100 μm). Safety assessments at 28 days show **(L-O)** serum uric acid, urea nitrogen, gamma-glutamyltransferase, and aspartate aminotransferase within normal limits without CSAδ-related elevations, and **(P)** hematoxylin–eosin sections from heart, liver, spleen, lung, and kidney reveal preserved architecture without overt pathology in CSAδ compared with saline or sham (scale bars, 100 μm). Data are mean ± SD (n = 3). One-way ANOVA with Tukey’s post hoc test; *p < 0.05, **p < 0.01, ***p < 0.005; ns, not significant. **Abbreviations.** WGA, wheat germ agglutinin; cTnI, cardiac troponin I; DAPI, 4′,6-diamidino-2-phenylindole; MDA, malondialdehyde; SOD, superoxide dismutase; IL-6, interleukin-6; IGF-1, insulin-like growth factor-1; LVAWd, left ventricular anterior wall thickness at end-diastole; CK-MB, creatine kinase-MB; APD90, action potential duration at 90% repolarization; CTD90, Ca²⁺ transient duration to 90% recovery; H&E, hematoxylin and eosin; IF, immunofluorescence.


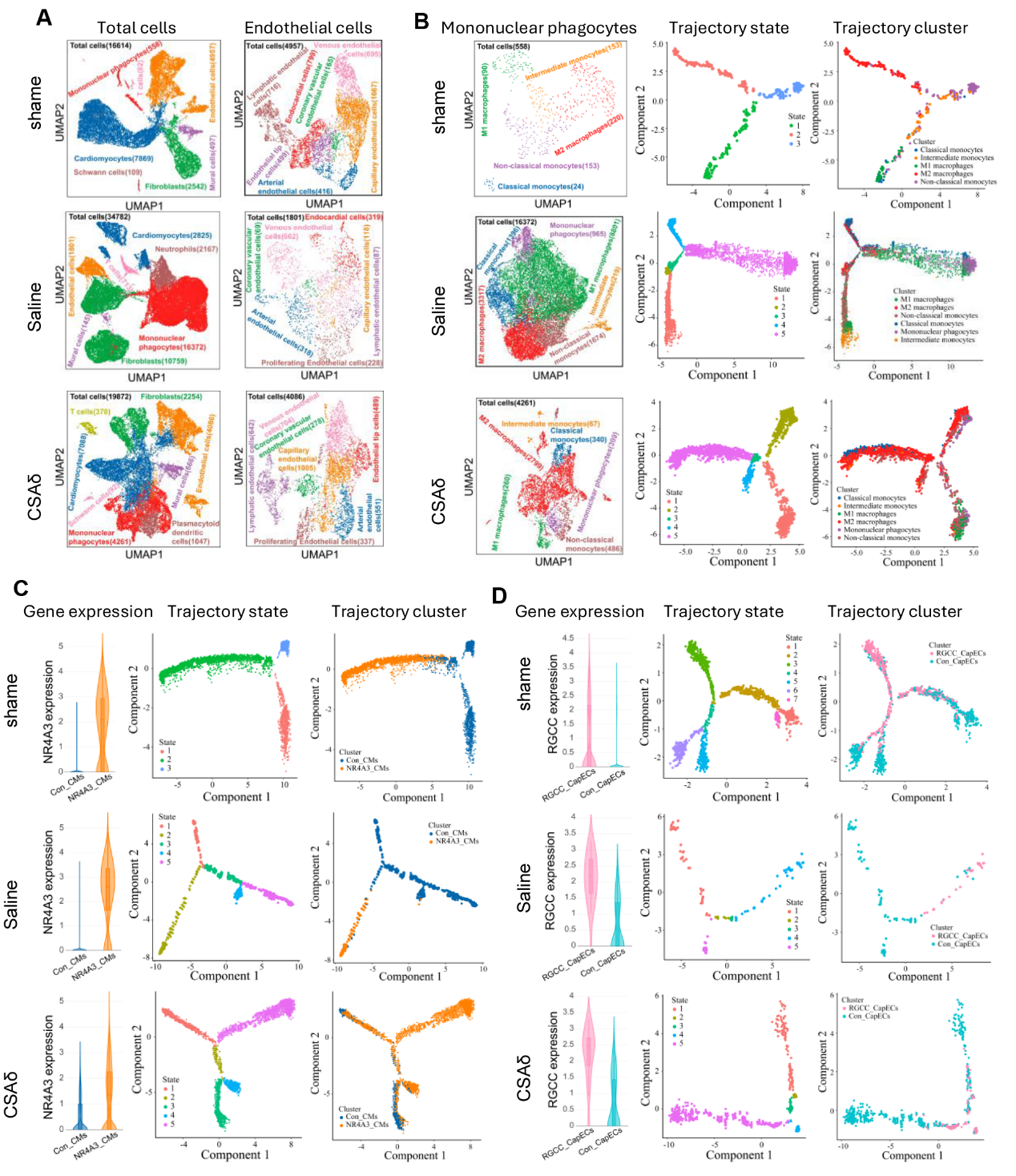


**Figure S10. Atlas and trajectories in aged pig MIR. (A)** UMAPs of total nuclei (left) and endothelial subtypes (right) for Sham, Saline, and CSAδ, showing broad lineage composition and EC diversity. **(B)** Mononuclear phagocytes: UMAP re-clustering (left) with trajectory state (middle) and trajectory cluster (right) inference. MIR drives differentiation toward M1 macrophages with an early-state M1 branch; CSAδ redirects trajectories toward M2 macrophages. **(C)** NR4A3_CMs: violin plots (normalized expression), trajectory states, and trajectory clusters indicate these CMs occupy early-intermediate pseudotime; CSAδ preserves this node compared with depletion in Saline. **(D)** RGCC_CapECs: analogous analyses show early–intermediate positioning and CSAδ-mediated rescue.
Trajectory were inferred from the integrated manifold with standard lineage-aware methods; plots display component space, state ordering, and cluster annotations to visualize branch progression.


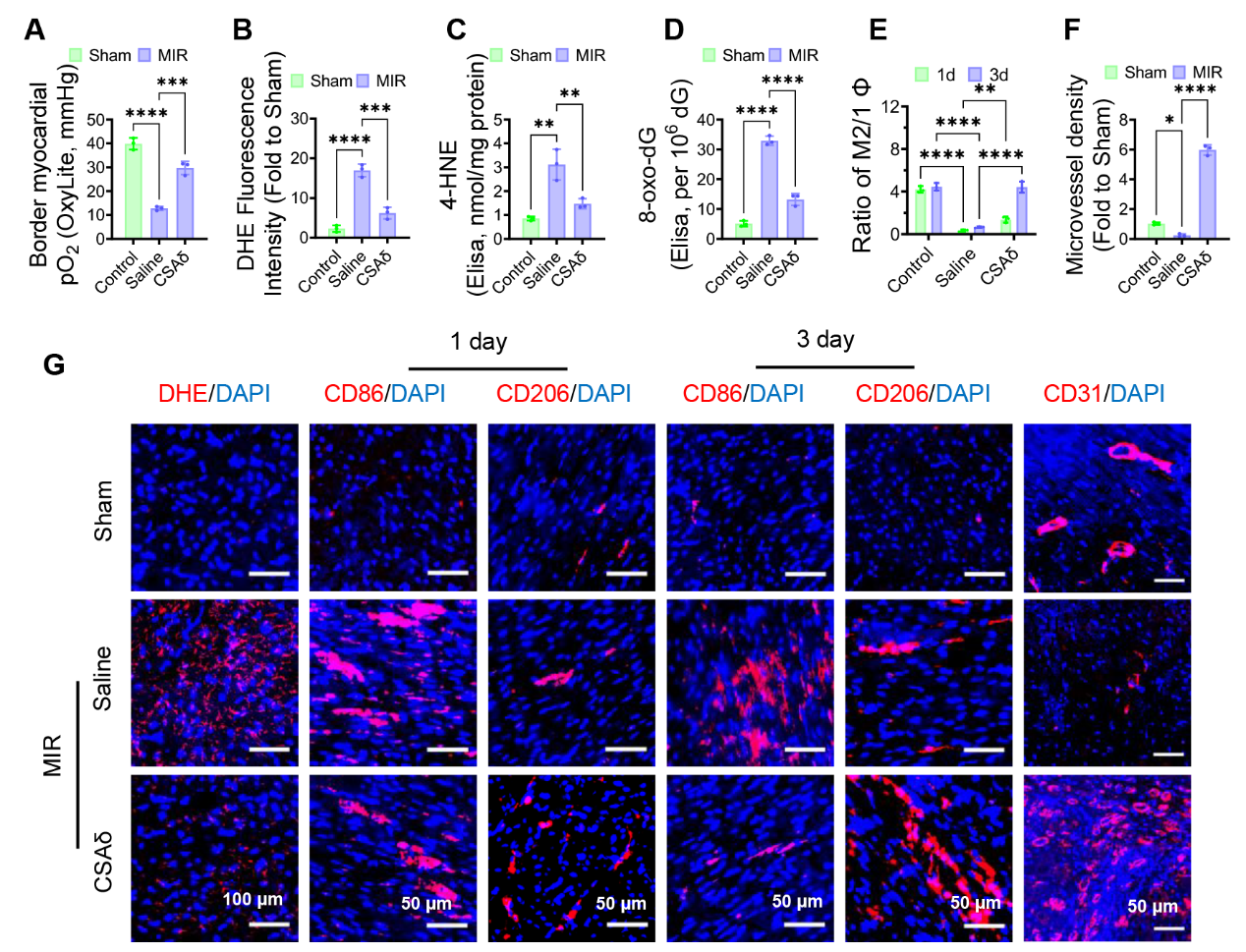


**Figure S11. CSAδ normalizes oxygenation, redox, immunity, and angiogenesis in aged pig MIR. (A)** In situ OxyLite measurements show higher border-zone pO₂ (mmHg) with CSAδ, indicating improved local oxygenation after reperfusion. **(B-D)** Oxidative stress is attenuated by CSAδ: DHE fluorescence (expressed as fold change versus Sham) is reduced, and ELISA quantification shows lower 4-HNE and 8-oxo-dG, consistent with diminished lipid peroxidation and DNA oxidation. **(E)** The M2/M1 macrophage ratio increases at day 1 and day 3 with CSAδ, indicating early immunomodulation toward a reparative phenotype. **(F)** CD31⁺ microvessel density (fold to Sham) rises with CSAδ, supporting enhanced angiogenic remodeling. **(G)** Representative immunofluorescence images, DHE/DAPI, CD86/DAPI (M1), CD206/DAPI (M2) at day 1 and day 3, and CD31/DAPI, illustrate reduced ROS signal, fewer M1 cells, more M2 cells, and denser capillary staining in CSAδ-treated myocardium (scale bars, 100 μm for DHE and 50 μm for others). Data are mean ± SD (n = 3). One-way ANOVA with Tukey’s post hoc test; *p < 0.05, **p < 0.01, ***p < 0.005, ****p < 0.001; ns, not significant. **Abbreviations.** pO₂, partial pressure of oxygen; DHE, dihydroethidium; 4-HNE, 4-hydroxynonenal; 8-oxo-dG, 8-oxo-2′-deoxyguanosine; M1/M2, pro-inflammatory/repair macrophages; IF, immunofluorescence.
